# GWAS SNPs Impact Shared Regulatory Pathways Amongst Multimorbid Psychiatric Disorders and Cognitive Functioning

**DOI:** 10.3389/fpsyt.2020.560751

**Published:** 2020-10-23

**Authors:** Evgeniia Golovina, Mark H. Vickers, Christopher D. Erb, Justin M. O'Sullivan

**Affiliations:** ^1^Liggins Institute, University of Auckland, Auckland, New Zealand; ^2^A Better Start National Science Challenge, Auckland, New Zealand; ^3^School of Psychology, University of Auckland, Auckland, New Zealand

**Keywords:** attention deficit hyperactivity disorder, anxiety, bipolar disorder, schizophrenia, unipolar depression, cognitive functioning, multimorbidity, p-factor

## Abstract

**Background:** Epidemiological research has reported that attention-deficit hyperactivity disorder (ADHD), anxiety, bipolar disorder (BD), schizophrenia (SCZ), and unipolar depression (UD) are multimorbid conditions that are typically accompanied by cognitive advantages or deficits, suggesting that common biological mechanisms may underlie these phenotypes. Genome-wide association studies (GWAS) have identified single-nucleotide polymorphisms (SNPs) associated with psychiatric disorders and cognitive functioning. However, the mechanisms by which these SNPs contribute to multimorbidities amongst psychiatric and cognitive phenotypes remains largely unknown.

**Objective:** To identify shared regulatory mechanisms amongst multimorbid psychiatric disorders and cognitive functioning.

**Methods:** We integrated data on 3D genome organization, expression quantitative trait loci (eQTLs), and pathway analyses to identify shared and specific regulatory impacts of 2,893 GWAS SNPs (*p* < 1 × 10^−6^) associated with ADHD, anxiety, BD, SCZ, UD, and cognitive functioning on genes and biological pathways. Drug-gene interaction analysis was performed to identify potential pharmacological impacts on these genes and pathways.

**Results:** The analysis revealed 33 genes and 62 pathways that were commonly affected by tissue-specific gene regulatory interactions associated with all six phenotypes despite there being no common SNPs in our original dataset. The analysis of brain-specific regulatory connections revealed similar patterns at eQTL and eGene levels, but no pathways shared by all six phenotypes. Instead, pairwise overlaps and individualized pathways were identified for psychiatric and cognitive phenotypes in brain tissues.

**Conclusions:** This study offers insight into the shared genes and biological pathways that are affected by tissue-specific regulatory impacts resulting from psychiatric- and cognition-associated genetic variants. These results provide limited support for the “p-factor” hypothesis for psychiatric disorders and potential mechanisms that explain drug side-effects. Our results highlight key biological pathways for development of therapies that target single or multiple psychiatric and cognitive phenotypes.

## Introduction

Attention-deficit hyperactivity disorder (ADHD), anxiety, bipolar disorder (BD), schizophrenia (SCZ), and unipolar depression (UD) are highly prevalent psychiatric disorders (1). Epidemiological research has reported that ADHD (2), anxiety (2, 3), BD (2), SCZ (4), and UD (5) are highly heritable (6) and represent multimorbid conditions that can be accompanied by cognitive advantages or deficits (7). Consistent with this, multiple genetic studies of psychiatric disorders and cognitive functioning have revealed a surprising degree of genetic overlap amongst these phenotypes, with the highest genetic correlations observed between SCZ and BP (r_g_ = 0.70), ADHD and UD (r_g_ = 0.44), BP and UD (r_g_ = 0.36), SCZ and UD (r_g_ = 0.34) (8–10). Collectively, this suggests the possible existence of common biological mechanisms underlying these phenotypes. Recent studies have proposed the term p-factor to represent common features of psychiatric disorders and an individual's overall liability to psychopathology (11, 12). Epidemiological evidence on the persistence, co-occurrence, sequential psychiatric multimorbidities, and genomic research tends to support the p-factor hypothesis. However, how the genetic architectures of psychiatric phenotypes contribute to the psychiatric multimorbidity (i.e., the “p-factor”) and the relationship with cognitive functioning is incompletely understood (11, 12). Furthermore, assuming that common drug targets may trigger similar therapeutic effects that induce similar signaling pathways and, thus, similar side effects, understanding the p-factor will be useful for prognosing therapeutic approaches and predicting drug side-effects.

Genome-wide association studies (GWAS) have identified thousands of single nucleotide polymorphisms (SNPs) that are associated with psychiatric and cognitive phenotypes (6). The majority of these genetic variants are non-coding and are likely to be expression quantitative trait loci (eQTLs) associated with the regulation of gene expression (13, 14). However, identifying the functional impacts of these eQTL SNPs is a significant hurdle (15).

Tissue-specific regulatory regions harbor substantial genetic risk for complex phenotypes (16–18). As such, decoding tissue-specific regulatory networks is an important step toward understanding the molecular mechanisms underlying psychiatric and cognitive phenotypes and identifying novel pathway-based therapeutic strategies.

The three-dimensional (3D) organization of the genome includes cell-type and tissue-specific spatial interactions between regulatory regions (marked by eQTL SNPs) and the genes that they control (hereafter eGenes) (19). These spatial interactions include cis- (<1 Mb between regulatory region and target eGene) and trans-acting (>1 Mb) intra- and interchromosomal connections. Recent studies have demonstrated the potential of integrating phenotype-associated SNPs, genome structure, and eQTLs for the identification of functional regulatory interactions in SCZ (20, 21). However, if or how genetic variation impacts on the regulation of shared or specific biological pathways to contribute to the development of psychiatric and cognitive multimorbidities remains unknown.

In this study, we integrated data on 3D genome organization and eQTLs to identify the tissue-specific spatial regulatory impacts of SNPs associated with cognitive functioning and five psychiatric disorders (i.e., ADHD, anxiety, BD, UD, SCZ). We hypothesized that this approach would enhance the discovery of shared characteristics (i.e., eGenes and biological pathways) among psychiatric and cognitive phenotypes, thereby potentially disclosing the mechanism for the p-factor that contributes to the observed multimorbidities.

## Materials and Methods

### Ethical Approvals for Data Access

Data access approval was obtained from dbGaP (*https://www.ncbi.nlm.nih.gov/gap/*) for Hi-C data sets for HeLa (project #18446: “Finessing predictors of cognitive development (part 2)”), cortical plate and germinal zone neurons (project #16489: “Finessing predictors of cognitive development”).

### Identification of Psychiatric and Cognition-Associated SNPs

Single-nucleotide polymorphisms (SNPs) associated (suggestive *p* < 1 × 10^−6^ cut-off was used) with ADHD, anxiety, BD, UD, SCZ, and cognitive functioning were downloaded from the GWAS Catalog (*www.ebi.ac.uk/gwas/*; 07/12/2018 and 14/07/2018; Supplementary Spreadsheet 1). Nine cognitive traits (i.e., intelligence, information processing speed, cognition, reading ability, reasoning, mathematical ability, infant expressive language ability, language performance, and speech perception) were combined to create the “cognitive functioning” category. Genomic positions and annotations of SNPs were obtained for the human genome build hg19 release 75 (GRCh37). To obtain genomic region information for SNPs, functional SNP annotation was performed using wANNOVAR (22, 23) (*http://wannovar.wglab.org/*).

### Hi-C Data Processing

Three-dimensional (3D) chromatin interactions in the nucleus can bring genes and distant regulatory elements into close spatial proximity and, thus, significantly influence gene expression regulation. Chromosome Conformation Capture (3C)-based technologies, such as Hi-C, capture these chromatin interactions in a genome-wide fashion and enable the investigation of the relationship between genome organization and genome activity. In order to study spatial tissue-specific regulatory interactions, we analyzed 28 cell type and tissue-specific Hi-C chromatin interaction libraries, including high-resolution data (24) and brain specific datasets (19, 25, 26) (Supplementary Table 1). Hi-C interaction data were downloaded from GEO (*https://www.ncbi.nlm.nih.gov/geo/*). Raw data were analyzed according to Rao et al. (24) [Juicer (27), version 1.5, *https://github.com/aidenlab/juicer*] to generate Hi-C libraries. This analysis pipeline included BWA alignment of paired-end reads onto the hg19 reference genome (BWA, version 0.7.15), merging paired-end read alignments, and removing chimeric, unmapped and duplicated reads. The remaining read pairs we refer to as “contacts.” Only Hi-C libraries that contain >90% alignable unique read pairs, and >50% unique contacts (<40% duplication rate) within the total sequenced read pairs were included in the analysis. Files containing cleaned Hi-C contacts locations (i.e., ^*^_merged_nodups.txt files) were processed to obtain Hi-C chromatin interaction libraries in the following format: read name, str1, chr1, pos1, frag1 mapq1, str2, chr2, pos2, frag2, mapq2 (str = strand, chr = chromosome, pos = position, frag = restriction site fragment, mapq = mapping quality score, 1 and 2 correspond to read ends in a pair). Reads where both ends had a mapq ≥ 30 were included in the final library. Hi-C chromatin interactions represent all captured pairs of interacting restriction fragments in the genome (Supplementary Figure 1). As such, restriction fragments were used to identify regulatory interactions between SNPs and genes.

### Identification of Spatial Regulatory Interactions Using CoDeS3D

CoDeS3D (28) (*https://github.com/Genome3d/codes3d-v1*) was used to identify spatial regulatory interactions between genes and phenotype-associated SNPs (Supplementary Figure 1). Briefly, the reference genome (hg19) was digested using the restriction enzyme that was used in the Hi-C library preparation (i.e., Mbol or HindIII). The restriction fragments containing phenotype-associated SNPs were identified. Next, the algorithm captured the restriction fragments interacting with the SNP-containing fragments in each of 28 Hi-C chromatin interaction libraries. Only interactions between SNP-containing fragments and restriction fragments overlapping a gene (defined using GENCODE transcript model version 19) were further analyzed (hereafter SNP-gene pairs). Next, the GTEx database (*https://gtexportal.org/*, GTEx multi-tissue dataset v7, Supplementary Table 2) was queried to identify only those spatial SNP-gene pairs, where expression levels of the gene (i.e., eGene) were associated with a SNP (i.e., eQTL SNP) in one or more of 48 tissues. Lastly, significant eQTL SNP-eGene-tissue interactions were identified using the Benjamini-Hochberg FDR control algorithm (29) to adjust eQTL *p*-values (FDR < 0.05). The FDR correction was performed across all tissue types. Significant eQTL-eGene interactions were defined as brain-specific if: (1) the spatial interaction was present in one or more brain-specific Hi-C dataset; and (2) the eQTL occurred in brain and spinal cord tissues.

### Bootstrapping Analysis

We performed bootstrapping (*N* = 10,000 iterations) with two reference eGene sets to test if the observed spatial eGene associations and overlaps were non-random. Reference set 1 contained the list of all genes in the genome (GENCODE transcript model version 19). Reference set 2 included the genes that were identified as interacting with the phenotype associated SNP containing fragments within the Hi-C libraries, to account for any potential bias in Hi-C gene coverage (i.e., the output of the find_genes module). Each bootstrapping iteration generated samples containing an equivalent number of randomly selected eGenes, as identified for each of the phenotypes being tested. The number of shared eGenes amongst phenotypes was counted for each bootstrap iteration. After 10,000 iterations we counted those instances where the number of shared eGenes in the bootstrapped overlap (*eGenes_bootstrapped*) is greater than or equal to the number of shared eGenes in the observed overlap (*eGenes_observed*). The *p*-value was calculated as the sum of these instances divided by the total number of iterations *N*,

(1) $$\begin{array}{l} p\;value=\frac{\sum \left(eGenesbootstrapped\;\geq \;eGenesobserved\right)}{N} \end{array}$$

For bootstrapping, if the *p* < 0.01 we reject the null hypothesis (i.e., the assumption that the observed eGene overlap is due to chance) and accept the alternative hypothesis that the observed relationship is non-random.

### Functional Enrichment Analysis

Functional gene enrichment analysis was performed using the g:GOSt tool from g:Profiler (30) (*https://biit.cs.ut.ee/gprofiler/*). The g:GOSt tool maps genes to known functional annotations and identifies the most impacted and statistically significant enriched annotations. eGene enrichment was tested within the biological process, molecular function, and cellular component gene ontology terms. All known human genes were chosen as the statistical domain scope. The significance of the overrepresented GO terms was corrected using the SCS algorithm (30) (adjusted *p* < 0.05).

### Pathway Analysis

eGenes were analyzed using iPathwayGuide (*https://www.advaitabio.com/ipathwayguide*, 09/13/2019) to identify enriched biological pathways. iPathwaysGuide uses the KEGG pathways database (31) and considers the role, position and relationships of each gene within a pathway to significantly reduce the number of false positives and identify truly impacted pathways. The FDR algorithm (29) was applied to correct *p*-values for multiple testing and determine significance at the pathway level (FDR < 0.05).

As for the gene analysis, bootstrapping (*N* = 10,000 iterations) was performed to determine the significance of the observed pathway overlaps. Here the KEGG pathways database (31) (*n* = 536; iPathwaysGuide, 22/01/2020) was used as the reference set.

### Correlation Analysis

A Pearson's correlation analysis was performed to measure the association between GTEx tissue sample size and the number of cis- and trans-acting eQTL-eGenes interactions.

### Drug-eGene Interaction Analysis

The Drug Gene Interaction database (32) (DGIdb, v3.0.2—sha1 ec916b2, *http://www.dgidb.org/*) consolidates and organizes known drug-gene interactions and gene druggability information from 30 databases and clinical trials. The collapsed resources include: DrugBank, PharmGKB, ChEMBL, FDA Biomarkers, Guide To Pharmacology, Jax-Clinical Knowledgebase, TDG Clinical Trials, CIViC, and CancerCommons. DGIdb supports over 40,000 genes and 10,000 drugs that are involved in over 15,000 drug-gene interactions. We queried DGIdb to identify drugs (their effects and mechanisms of action) that target the eGene products.

## Results

### SNPs Associated With Psychiatric and Cognitive Phenotypes Regulate Distant Genes

**Figure 1:**
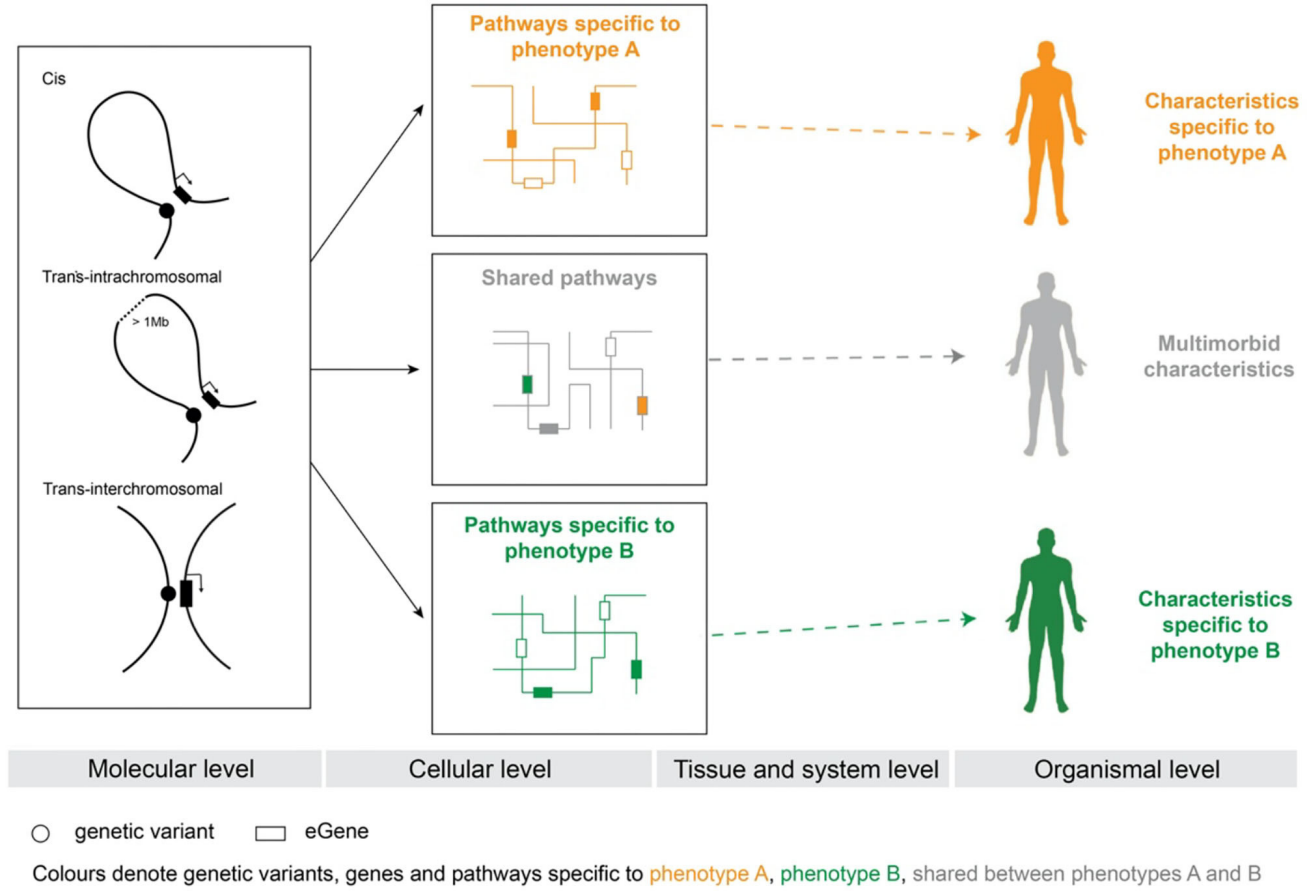
Genetic model of multimorbidity and the SNP-phenotype relationship. Phenotype-specific genetic variants alter tissue-specific gene expression by changing regulatory connections within the 3D dimensional organization of the genome. The gene products, whose expression is altered, interact within biological pathways. Multimorbidity results when affected gene products co-occur within pathways. The co-occurrence of affected gene products within shared pathways changes the way pathways respond to environmental signals and thus affects cellular activities at tissue and system levels. Orange—genetic variants, genes, and pathways specific to phenotype A. Green—genetic variants, genes, and pathways specific to phenotype B. Gray—genetic variants, genes, and pathways shared between phenotypes A and B. White—genes that are not specific to either phenotype A or phenotype B.

**Figure 2:**
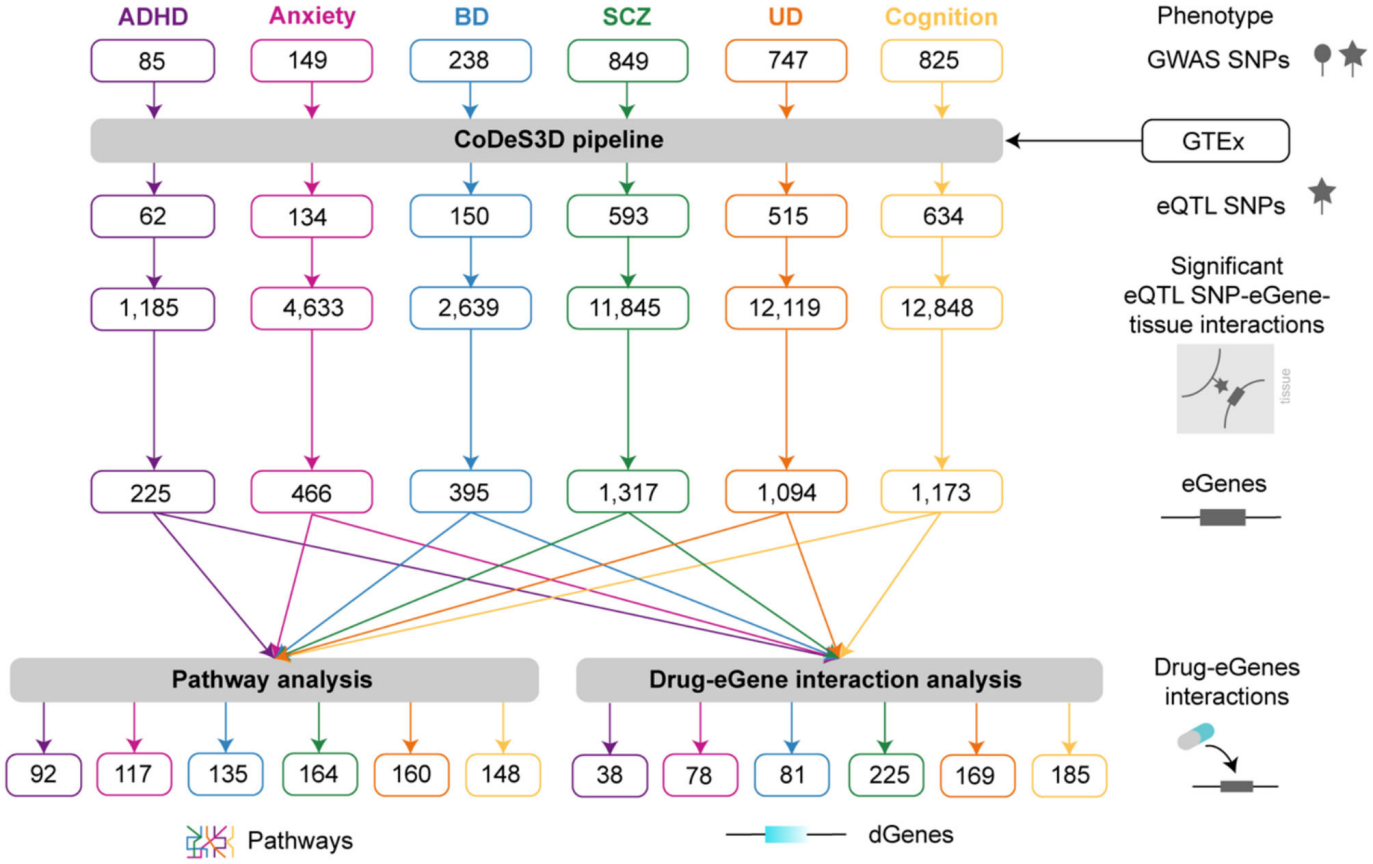
Pipeline used to study the multimorbidities between psychiatric and cognitive phenotypes. SNPs associated with ADHD, anxiety, BD, SCZ, UD, and cognitive functioning were obtained from the GWAS Catalog and analyzed using CoDeS3D (Supplementary Figure 1) to identify the genes associated with significant spatial eQTLs. Phenotype-specific lists of eQTLs are presented in Supplementary Spreadsheet 2. Pathway analysis was used to identify pathways containing co-occurring eGenes for the different phenotypes (Supplementary Table 5). Drug-eGene interaction analysis was performed to identify druggable genes (Supplementary Spreadsheet 5).

We hypothesized that multimorbidity is driven by genetic variants (e.g., SNPs, structural variants, indels) that regulate tissue-specific expression of genes that co-occur within specific biological pathways and thus affect the phenotype (Figure 1). CoDeS3D (28) was used to integrate genome structure and eQTL data to identify tissue-specific spatial eQTLs for SNPs (*n* = 2,893) associated (*p* < 1 × 10^−6^) with cognitive functioning, ADHD, anxiety, BD, UD, and SCZ. We identified a total of 45,269 significant tissue-specific eQTL-eGene pairs (FDR < 0.05) from 48 different human tissues (Figure 2, Supplementary Spreadsheet 2). In total, 2,088 SNPs were identified as eQTLs (Figure 2). Approximately 60% of the SNPs associated with each phenotype were eQTLs (Supplementary Figure 2A). The majority of the 2,088 eQTLs we identified were located within introns and intergenic regions (Supplementary Figure 2B). The patterns of cis- and trans-acting regulatory interactions formed by eQTLs within introns and intergenic regions were similar across all phenotypes (Supplementary Figures 3A,B).

### SNPs and eQTLs Are Mostly Unique to Individual Phenotypes

**Figure 3:**
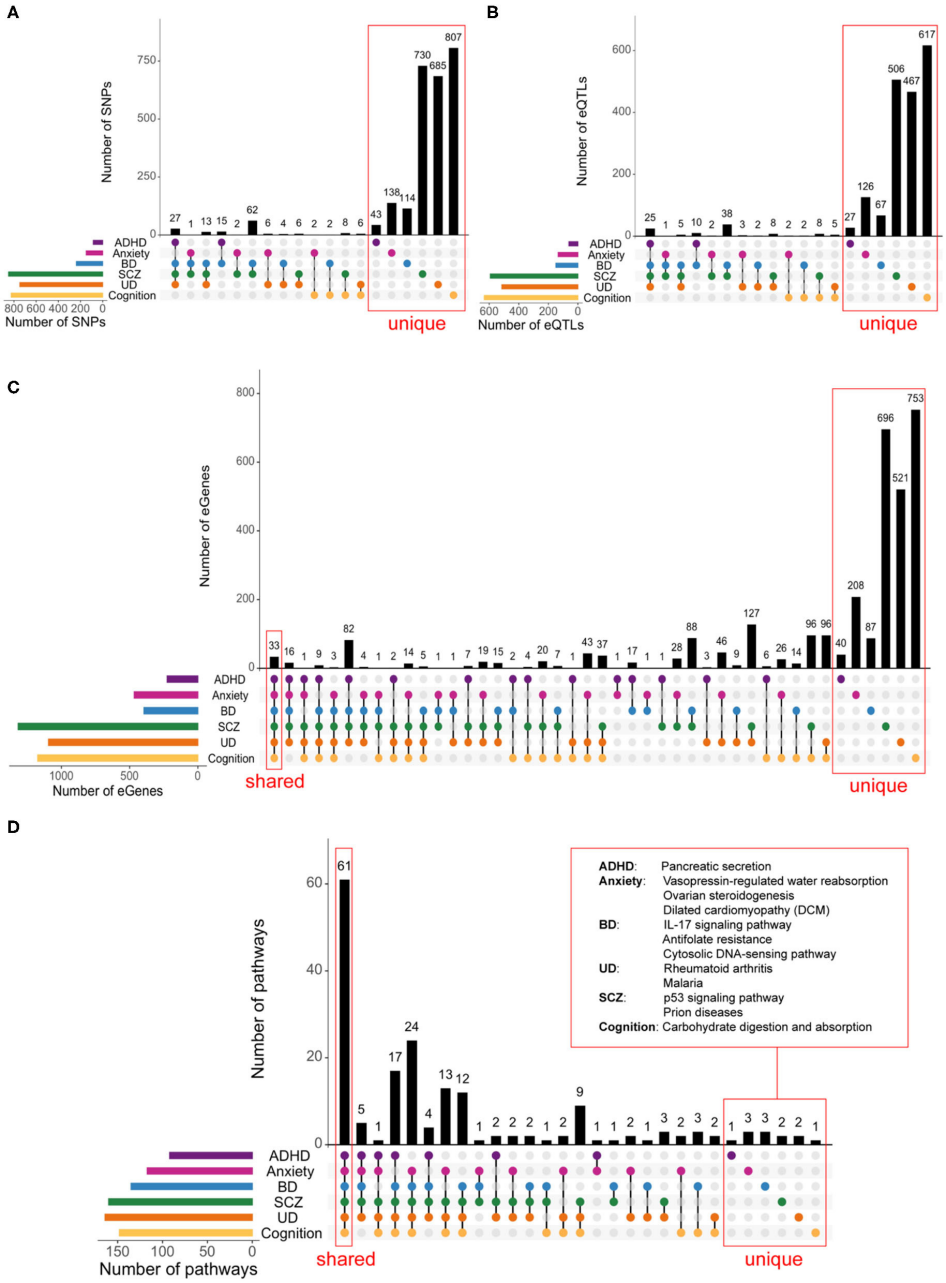
Shared biological pathways link psychiatric disorders and cognition. Psychiatric disorders and cognitive functions have low levels of genetic similarity at the SNPs **(A)**, eQTLs **(B)**, and eGene **(C)** levels. A FDR-adjusted *p* < 0.05 was used to identify eQTLs and eGenes. **(D)** Psychiatric disorders and cognition share a large degree of commonality at the biological pathways level. Biological pathways containing eGenes for each phenotype were identified using iPathwayGuide (Supplementary Spreadsheet 4). Among the most impacted pathways, 61 were shared between psychiatric disorders and cognition, 66—across all five psychiatric disorders. Only one pathway (i.e., Pancreatic secretion) was unique to ADHD, three pathways [i.e., Vasopressin-regulated water reabsorption, Ovarian steroidogenesis, and Dilated cardiomyopathy (DCM)] were specific to anxiety, three were unique to BD (i.e., IL-17 signaling pathway, Antifolate resistance, and Cytosolic DNA-sensing pathway), 2—to SCZ (i.e., p53 signaling pathway and Prion diseases), 2—to UD (i.e., Rheumatoid arthritis and Malaria) and 1—to cognition (i.e., Carbohydrate digestion and absorption). The full list of shared and unique pathways is in Supplementary Spreadsheet 5.

Previous studies have described a limited number of SNPs associated with combined phenotypes [i.e., SCZ+cognition (7), BD+cognition (7) and BD+SCZ (33)]. Therefore, we intersected the SNP and spatial eQTL sets associated with ADHD, anxiety, BD, UD, SCZ, and cognitive functioning to identify shared genetic variation between these phenotypes. We found that the total numbers of shared SNPs across phenotype combinations are similar to those for shared eQTLs (Figures 3A,B). No SNPs, or eQTLs were common to all phenotypes (Figures 3A,B, respectively). However, there were limited eQTL overlaps amongst the psychiatric disorders and between psychiatric disorders and cognition (Figure 3B). We did not observe any overlapping eQTLs for ADHD and cognitive phenotypes. The limited SNP and eQTL overlaps we observed supports the hypothesis that regulatory impacts on genes and pathways can make a contribution to multimorbidity.

### Psychiatric and Cognitive Phenotypes Share Common eGenes

Multimorbidities among psychiatric disorders and cognition could result from the regulatory effects of shared or phenotype-specific eQTLs on shared gene targets. Shared eGenes (*n* = 33) were identified for eQTLs associated with all six phenotypes (Figure 3C). SCZ and UD shared the greatest number of eGenes (*n* = 374). ADHD and cognition have 58 shared eGenes despite having no shared eQTLs (Figures 3B,C). Bootstrapping simulations (*n* = 10,000) confirmed that the overlaps were significant (*p* < 0.001; Supplementary Figure 4A). The expression levels of the 33 eGenes (Supplementary Table 3) that were shared across all phenotypes are associated with eQTLs that are located within three loci (chr3:52256696-53455568, chr6:25177507-32914725, and chr10:103816827-105039240; Supplementary Figure 5).

Functional assignments for phenotype-associated SNPs are typically made to the closest gene to the phenotype-associated variant (9, 21, 34–36). Our analysis of eQTL-eGenes spatial connections showed that 5–7% are explained by associations with the closest gene (Supplementary Spreadsheet 2). For example, ADHD, BD, UD, and SCZ (34) -associated rs2535629, which is located within an intron of *ITIH3*, is an eQTL for *ITIH4* in the brain, adipose tissues, and the cardiovascular system. However, rs2535629 is also associated with spatial regulatory interactions with another 12 eGenes (*ITIH3, PPM1M, GNL3, RAF1, MUSTN1, NEK4, NT5DC2, PBRM1, RBMS3, SFMBT1, TMEM110, WDR82;* Supplementary Table 4). Thus, incorporating data on spatial genome organization enables the identification of local and distal eQTL-gene connections that can potentially contribute to multimorbid phenotypes.

### Psychiatric and Cognitive Phenotypes Share Common Biological Pathways

The pleiotropic effects of eQTLs and co-occurrence of the affected eGenes within biological processes and pathways could contribute to the underlying multimorbid conditions (Figure 1). Gene ontology (GO) analysis of the 33 shared eGenes identified significant enrichment (adjusted *p* < 0.05) in gene expression, transcription, metabolic, biosynthetic, and regulatory processes (Supplementary Figure 6). Notably, ontological analyses of eGenes specific for each phenotype revealed associations with neurodevelopment (e.g., “nervous system development”), immune system processes (e.g., “immune response”), responses to environmental stimuli, and signal transduction that were common to all phenotypes (Supplementary Spreadsheet 3).

Pathway analysis using eGenes (shared and specific for each phenotype) identified 61 biological pathways that are shared by all phenotypes (Figure 3D). These shared pathways are associated with human diseases, signal transduction, neurodevelopment, learning, and immunity (Supplementary Spreadsheet 4, Supplementary Table 5). Bootstrapping (*n* = 10,000) confirmed that the observed overlap was highly significant (*p* < 0.001; Supplementary Figure 7) and not an artifact of the presence of shared eGenes within these pathways (Supplementary Figure 8). Notably, despite a high prevalence of comorbidity and substantial genetic correlation between anxiety and UD, only 6 pathways were shared between these two conditions (37–39).

**Figure 4:**
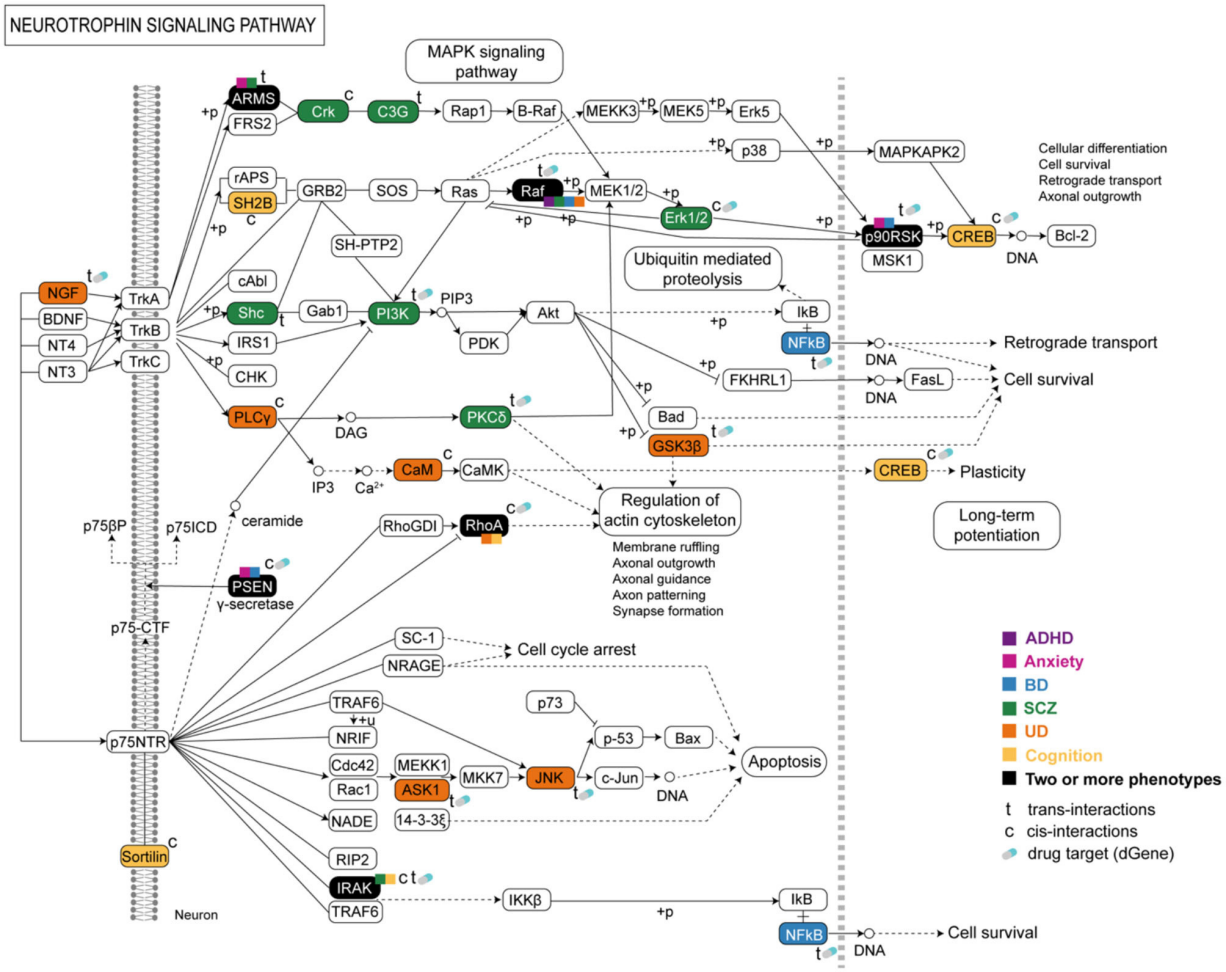
Psychiatric and cognitive SNPs mark eQTLs that are associated with gene expression within the neurotrophin signaling pathway. The co-occurrence of the affected shared or phenotype-specific eGenes and imbalance in gene expression within this pathway may lead to a series of cellular functions and events associated with psychiatric and cognitive phenotypes and the multimorbidities between them.

The neurotrophin signaling pathway is important in developmental neurobiology (40). This pathway contained eGenes associated with eQTLs from all phenotypes (Figure 4). Most of the eGenes within the neurotrophin signaling pathway were regulated by trans-acting eQTLs (Figure 4). Dysregulation in the neurotrophin signaling cascade can impact downstream pathways, e.g., axon guidance and long-term potentiation pathways, which also contained co-occurring eGenes associated with cognition and psychiatric phenotypes (Supplementary Figures 9, 10).

We also identified 12 biological pathways that were impacted by eGenes that were specific to individual psychiatric and cognitive phenotypes (Figure 3D, Supplementary Table 5).

### Drug-Gene Interactions Identify Pharmaceutical Impacts on Multimorbidity

Drugs to prevent, stabilize, or slow the progression of psychiatric and cognitive conditions often have side effects consistent with known multimorbidities (41). Between 15–21% of the eGenes we identified encode proteins that are targeted by existing drugs (Supplementary Spreadsheet 5). Four eGenes (i.e., *AS3MT, FLOT1, HLA-A*, and *PBRM1*) are affected by eQTLs from all tested phenotypes and are current therapeutic targets for psychiatric disorders (Supplementary Figure 11). For example, everolimus targets PBRM1 (polybromo 1 protein, subunit of chromatin remodeling complex) that can be important in neural development (42). Notably, everolimus improves memory and learning but simultaneously aggravates depression and anxiety in mice (43). Therefore, we contend that these observations provide insights into potential mechanisms for drug side-effects associated with multimorbidities among psychiatric disorders and cognition.

### Spatial Regulatory eQTL Effects Are Tissue-Specific

Brain structural changes are commonly considered to be relevant to the development of psychiatric and cognitive phenotypes. However, these phenotypes are also associated with physiological changes at the level of the whole body [e.g., with impaired functioning of endocrine (44), immune (45, 46), and cardiometabolic (47) systems]. Therefore, the tissue-specificity of phenotype-associated spatial regulatory impacts may provide important insights into psychiatric and cognitive multimorbidities. The cis- and trans-acting eQTLs we identified were widely distribution across human tissues (Supplementary Figure 12). The numbers of cis and intra-chromosomal spatial eQTL SNP-eGene regulatory interactions correlated with the GTEx tissue sample size (Supplementary Figure 13). By contrast, inter-chromosomal eQTLs were not correlated with tissue sample size (Supplementary Figure 13). We detected greater numbers of cis (<1 Mb) and intra-chromosomal (≥1 Mb) eQTL-eGene interactions in thyroid and brain cerebellum tissues, than were predicted by the correlation curve (Supplementary Spreadsheet 6, Supplementary Figure 13). This is consistent with reported roles for the thyroid in the development of cognitive functions and psychiatric disorders (44, 48).

### Brain-Specific Regulatory Associations Are Mostly Unique to Individual Phenotypes

**Figure 5:**
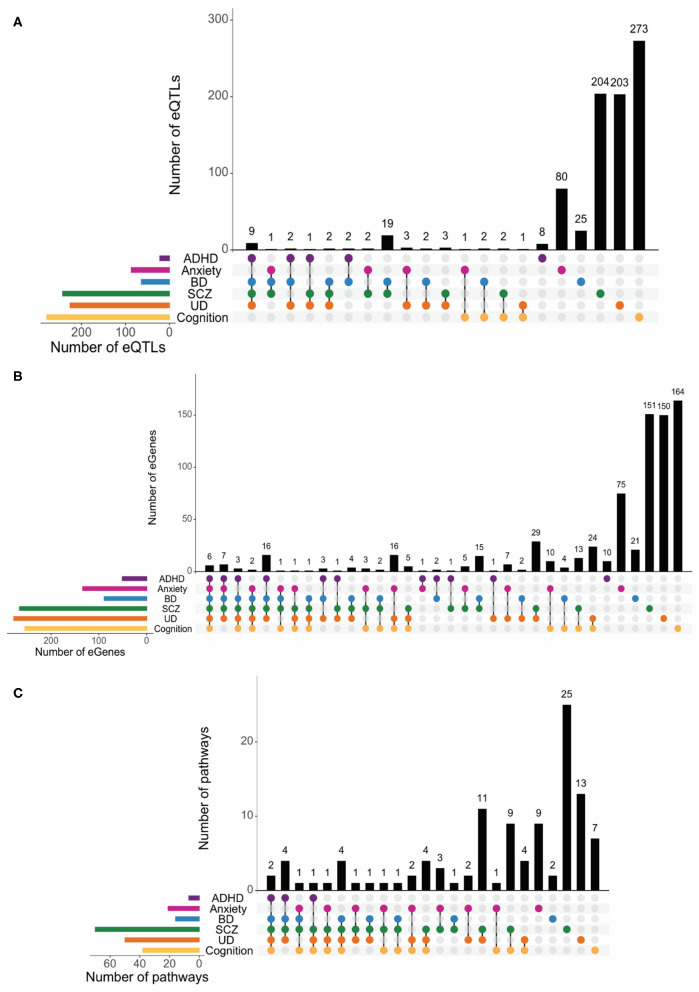
Psychiatric disorders and cognition show no shared pathways among all phenotypes in brain tissues. Psychiatric disorders and cognitive functions have low levels of genetic similarity at the eQTLs **(A)** and eGene **(B)** levels in brain tissues. A FDR-adjusted *p* < 0.05 was used to identify eQTLs and eGenes. More pairwise brain-specific pathway overlaps **(C)** and individualized pathways were identified for psychiatric and cognitive phenotypes.

ADHD, anxiety, BD, UD, SCZ, and cognitive functioning are widely regarded as brain-specific phenotypes. Therefore, we identified brain-specific eQTL-eGene regulatory interactions that are supported by spatial connections within brain-specific Hi-C datasets (Supplementary Spreadsheet 7). The brain-specific eQTLs and eGenes we identified were mostly unique to the individual psychiatric and cognitive phenotypes (Figures 5A,B). Consistent with our earlier findings across all tissues, we observed many shared pathways across different phenotype combinations. However, we observed more pairwise combinations and individualized pathways for the psychiatric and cognitive phenotypes in the brain (Figure 5C, Supplementary Table 6, Supplementary Spreadsheet 8). Whilst there were no brain-specific pathways that were shared across all phenotypes, the psychiatric disorders have between 6 and 24 shared pathways with the cognitive phenotypes (eTable 15 in Supplement 2). Again, ADHD was an exception and did not share any pathways with the cognitive phenotypes (Figure 5C). Bootstrapping (*n* = 10,000) confirmed the significance (*p* < 0.01) of the observed brain-specific eGene and pathway overlaps, except for the anxiety+BD+SCZ overlap (Supplementary Figure 14).

## Discussion

There is increasing evidence that psychiatric disorders and cognitive functioning share an underlying cause, the so-called “p factor” (11). The existence of the p factor is further supported by the identification of genetic overlaps between psychiatric disorders (12). Despite the growing recognition of the p factor for psychiatric conditions, how the p factor manifests biologically remains unclear. We hypothesized that the p factor results from the action of genetic variants associated with psychiatric and cognitive phenotypes on tissue-specific expression of genes within shared biological pathways. Consistent with our hypothesis, we identified relatively little common genetic risk at the level of SNPs and eQTLs, but extensive biological pathway overlaps. We identified 61 biological pathways that were shared amongst the tested psychiatric and cognitive phenotypes.

Even though the relevance of the pathways unique to individual psychiatric or cognitive phenotypes may not be immediately obvious (e.g., pancreatic secretion and ADHD), there is increasing evidence that psychiatric disorders represent complex multisystem conditions (49–51). Moreover, it is obvious that the brain is a component within a much larger system and is subject to both direct and indirect contributions from other tissues that impact psychiatric and cognitive phenotypes. This is epitomized by hypo and hyperthyroidism, whereby alterations to thyroid hormone status are associated with development of psychiatric conditions (e.g., SCZ, BD, anxiety and depression) (44, 52). Notably, we observed that the thyroid hormone signaling pathway was impacted by genetic variants specific to all six phenotypes.

Infections during pregnancy, at birth and in early childhood increase the risk of ADHD (53), BD (54), and SCZ (54). We identified changes to gene regulation within pathways associated with infectious diseases (e.g., HTLV-I infection, and hepatitis B) that involved eQTLs for all phenotypes. Phenotype specific overlaps in immune related pathways were retained in the brain specific analysis. For example, gene expression within the natural killer cell mediated cytotoxicity and Th1 and Th2 cell differentiation pathways was affected by genetic variants associated with ADHD, BD, UD, SCZ, and cognition. We also identified regulatory changes to genes within signaling and neurodevelopmental pathways. Collectively, these results are consistent with changes to gene regulation within signaling, immune, and neurodevelopmental pathways combining to affect the pathophysiology and development of ADHD, anxiety, BD, UD, SCZ, and their association with cognitive functions. Of course, the convergence of phenotype specific genetic impacts on shared biological pathways does not explain all the features of the observed multimorbidities between the tested phenotypes. Clearly, there remains a role for dysregulation within pathways unique to individual psychiatric and cognitive phenotypes. Moreover, it is important to remember that pathways represent part of many cell-type and tissue-specific networks and thus can exert their effects on the multimorbidity risk through these networks. The impacts of cell-type and tissue-specific networks were not explicitly considered in this study.

We identified drugs that target gene products encoded by genes impacted by eQTL SNPs associated with each of the tested psychiatric and cognitive phenotypes. We contend that the therapeutic targeting of shared genes helps to explain the psychiatric side effects that are observed for these drugs. This hypothesis could be tested by categorizing patients who exhibit treatment associated side-effects according to the presence or absence of the allele associated with the side-effect relevant phenotype. However, genes in the shared set can only be used in patient stratification for personalized treatment if the eQTL effects occur in the same direction. Arguably, these decisions will only affect clinical outcomes if the variants are of high penetrance. However, the validity of these assumptions remains to be determined.

The development of psychiatric and cognitive phenotypes depends on a complex, often non-linear, dynamic interplay between genetic and environmental factors. As such, we are aware that our study has several limitations. Firstly, increasing the number of GWAS studies on certain phenotypes (e.g., ADHD, anxiety, etc.) will result in the identification of additional novel SNP loci. Secondly, common SNPs do not explain all of the estimated heritability in psychiatric and cognitive phenotypes, suggesting that other factors (e.g., rare variants, indels, structural variation etc …) also contribute (55). Thirdly, the GTEx eQTL data used in this study was largely from European individuals aged 40 years and older (56). Thus, robust predictions of the regulatory mechanisms of psychopathology and cognitive functioning across different populations and developmental stages requires additional data sets. Moreover, the reduced numbers of brain samples may have made the eQTLs and thus pathway overlaps more difficult to detect, thus accounting for the observed reduction in phenotype overlap. Fourthly, SNPs associated with diverse cognitive functions were combined into one general “cognitive functioning” phenotype, and do not represent common factors underlying all cognitive traits (Supplementary Spreadsheet 1). As such further research is needed to look more precisely at specific aspects of cognitive functioning (e.g., mathematical ability, language performance etc…) and their relationship with psychiatric disorders. Fifthly, it should be noted that the way we identify eGenes (restriction fragments do not have to contain the eGene promoter) may lead to potential eGenes over-identification. Sixthly, we are also aware that there is a potential bias in the tools, datasets, and databases used in this study, therefore our findings do not necessarily represent the full picture. For example, the usage of Hi-C datasets for HeLa cells may introduce unwanted bias due to their aberrant karyotype. Moreover, our study of tissue-specific regulatory interactions was potentially confounded by the tissue specific expression and Hi-C datasets not being paired. This is particularly relevant if there is an interaction with RNA degradation during sample preparation. Tissue-specific eQTL SNP effects mediating the eGene overlaps may not be independent and represent side effects of chromatin-level regulation. Incorporating other tissue-specific multi-omics data [e.g., from ENCODE consortium (57)] could provide a more comprehensive and accurate identification of tissue-specific regulatory changes associated with psychiatric and cognitive phenotypes. Finally, despite the statistical significance of the observed enrichment (FDR < 0.05), it can be argued that these results are unrealistically positive and that a stricter cut-off (FDR < 0.01) should have been employed to further reduce the chances of identifying false positives. Despite these limitations, our analysis provides a starting point for further mechanistic and functional investigation. Replication analyses will increase the robustness of the results and provide a clearer indication of cross-phenotype overlap and phenotype-specific genetic architecture.

In conclusion, we have described biological pathways for multimorbidity and identified drug-gene interactions that may be clinically relevant for the treatment and prevention of psychiatric and cognitive phenotypes. Our results provide support for a shift from a gene-centric approach to identifying pathways for the treatment of multimorbid psychiatric and cognitive conditions. Future applications of the spatial genetic approach we used to other phenotypes will cross the molecular, cellular, tissue, and system levels to define personalized disease risk profiles, therapeutic targets and drug side-effects.

## Data Availability Statement

The datasets presented in this study can be found in online repositories. The names of the repository/repositories and accession number(s) can be found in the article/Supplementary Material.

## Author Contributions

EG performed the analyses and wrote the manuscript. MV co-supervised EG and commented on the manuscript. CE aided results interpretation and commented on the manuscript. JO'S supervised EG and co-wrote the manuscript. All authors contributed to the article and approved the submitted version.

## Conflict of Interest

The authors declare that the research was conducted in the absence of any commercial or financial relationships that could be construed as a potential conflict of interest.
