# Supplementary material for: GWAS SNPs Impact Shared Regulatory Pathways Amongst Multimorbid Psychiatric Disorders and Cognitive Functioning
Source: Front Psychiatry. 2020 Oct 23;11:560751. doi: 10.3389/fpsyt.2020.560751 (PMC7649776; doi:10.3389/fpsyt.2020.560751)
Supplement: Supplementary file 1 [file Data_Sheet_1.PDF]

Supplementary Material

GWAS SNPs Impact Shared Regulatory Pathways Amongst Multimorbid Psychiatric Disorders and Cognitive Functioning

Evgeniia Golovina[1,2], Mark H. Vickers [1], Christopher D. Erb[3], Justin M. O'Sullivan[1,2*]

[1]Liggins Institute, University of Auckland, Auckland, New Zealand

[2]A Better Start national Science Challenge, New Zealand

[3]School of Psychology, University of Auckland, Auckland, New Zealand

*** Correspondence:**
Justin M. O'Sullivan
justin.osullivan@auckland.ac.nz

1 Supplementary Methods

Source code for CoDeS3D pipeline is available at https://github.com/Genome3d/codes3d-v1

All python and R scripts used for data analysis and visualization are available at https://github.com/Genome3d/psychiatric_and_cognitive_multimorbidities.

R version 3.5.2 and RStudio version 1.1.463 were used for all R scripts. All python scripts are based on Python 2.7.15.

2 Supplementary Data

2.1 Supplementary Spreadsheet 1. GWAS SNPs associated with psychiatric disorders and cognitive functions (doi:10.17608/k6.auckland.12275747)

2.2 Supplementary Spreadsheet 2. Spatial eQTL SNP-eGene-tissue interactions associated with ADHD, anxiety, BD, UD, SCZ and cognitive functioning (doi:10.17608/k6.auckland.12276119)

2.3 Supplementary Spreadsheet 3. Gene Ontology enrichment analysis. Query sizes are 197 eGenes (ADHD), 395 eGenes (anxiety), 340 eGenes (BD), 930 eGenes (UD), 1109 eGenes (SCZ) and 1016 eGenes (cognitive functioning). Adjusted p < 0.05 (doi:10.17608/k6.auckland.12276203)

2.4 Supplementary Spreadsheet 4. Pathways associated with psychiatric disorders and cognitive functioning. pComb_FDR < 0.05 (doi:10.17608/k6.auckland.12276281)

2.5 Supplementary Spreadsheet 5. DGIdb drug-eGene interactions associated with psychiatric disorders and cognitive functioning (doi:10.17608/k6.auckland.12276383)

2.6 Supplementary Spreadsheet 6. Number of cis- (< 1Mb) and trans-acting (≥ 1Mb and interchromosomal) eQTL SNP-eGene interactions per GTEx tissue (GTEx v7) (doi:10.17608/k6.auckland.12276440)

2.7 Supplementary Spreadsheet 7. Brain-specific spatial eQTL SNP-eGene interactions associated with ADHD, anxiety, BD, UD, SCZ and cognitive functioning (doi:10.17608/k6.auckland.12276449)

2.8 Supplementary Spreadsheet 8. Brain-specific pathways associated with psychiatric disorders and cognitive functioning. pComb_FDR < 0.05 (doi:10.17608/k6.auckland.12276452)

3 Supplementary Figures and Tables

3.1 Supplementary Figures

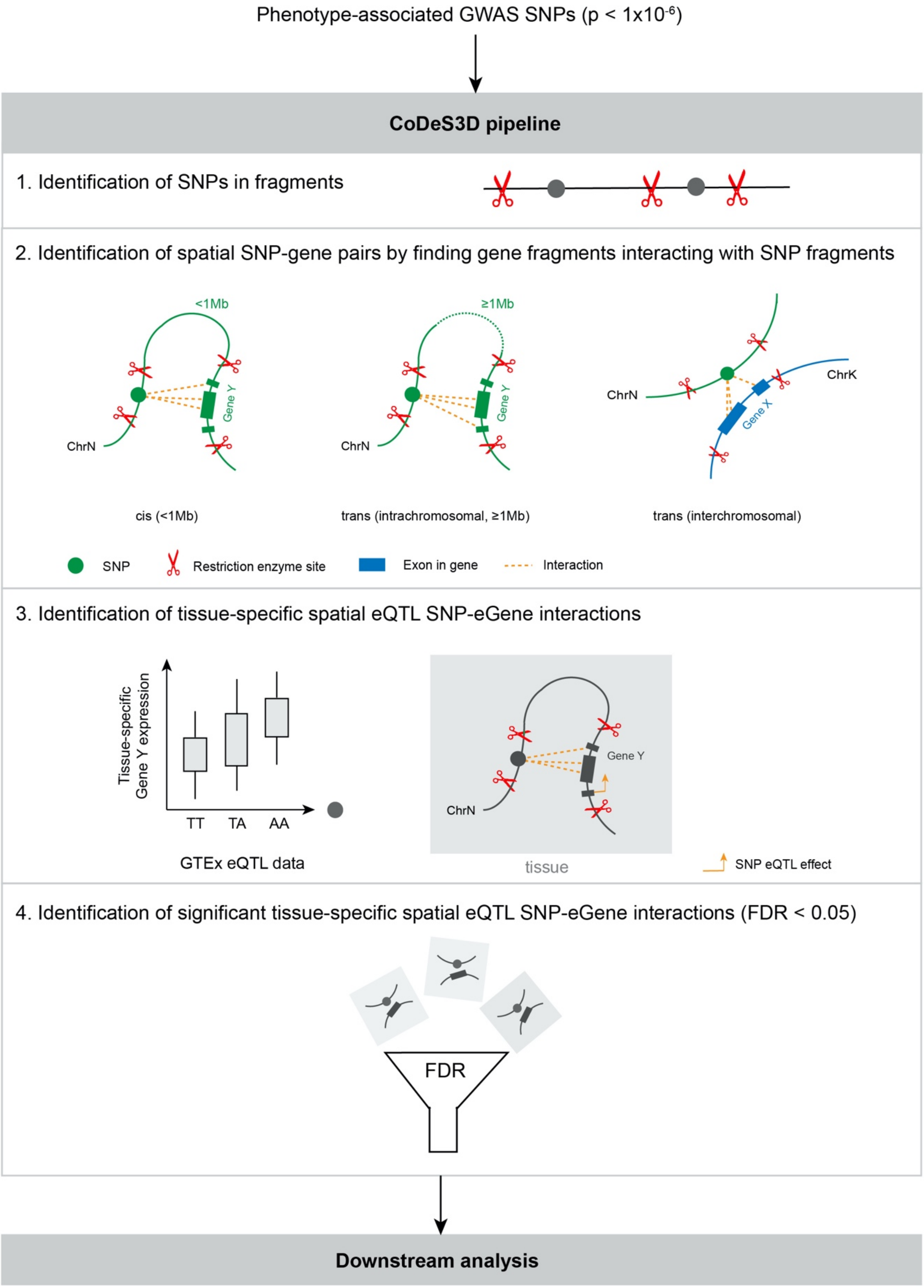

Supplementary Figure 1. The CoDeS3D pipeline used in this study. Restriction fragments containing SNPs associated with psychiatric disorders and cognition were identified. Hi-C libraries were interrogated to identify genes in fragments that spatially interact (in cis- and trans-) with SNP fragments. The resulting spatial SNP-gene pairs were used to query the GTEx database to determine functional tissue eQTL interactions between SNP and eGene (i.e. gene, whose expression is in eQTL with a SNP). Only statistically significant (FDR < 0.05) eQTL SNP-eGene-tissue interactions were further used in the downstream analysis (i.e. functional gene enrichment, pathway analysis, and drug-eGene interactions).

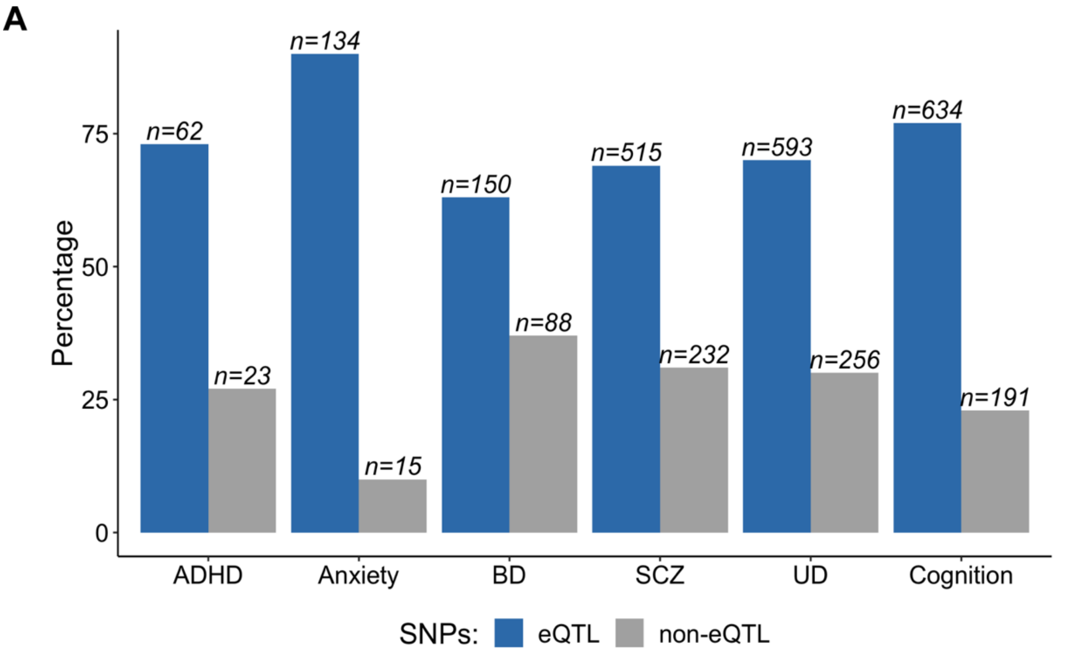

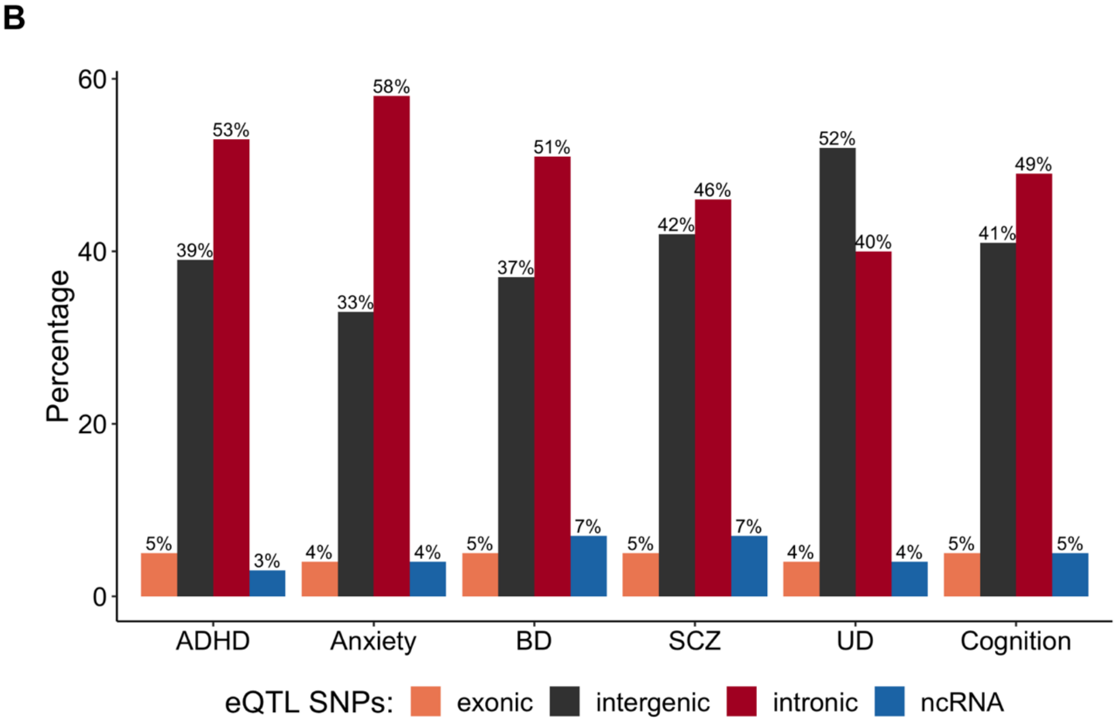

Supplementary Figure 2. The percentage contribution of GWAS SNPs (associated with cognitive and psychiatric phenotypes) that are correlated with mRNA expression of genes (i.e. eQTL SNPs) and their distribution in the genome. **A** The majority of the GWAS SNPs

associated with psychiatric disorders and cognition impact on gene expression as eQTLs. **B** Most of these eQTL SNPs fall within non-coding regions (i.e. introns and intergenic regions).

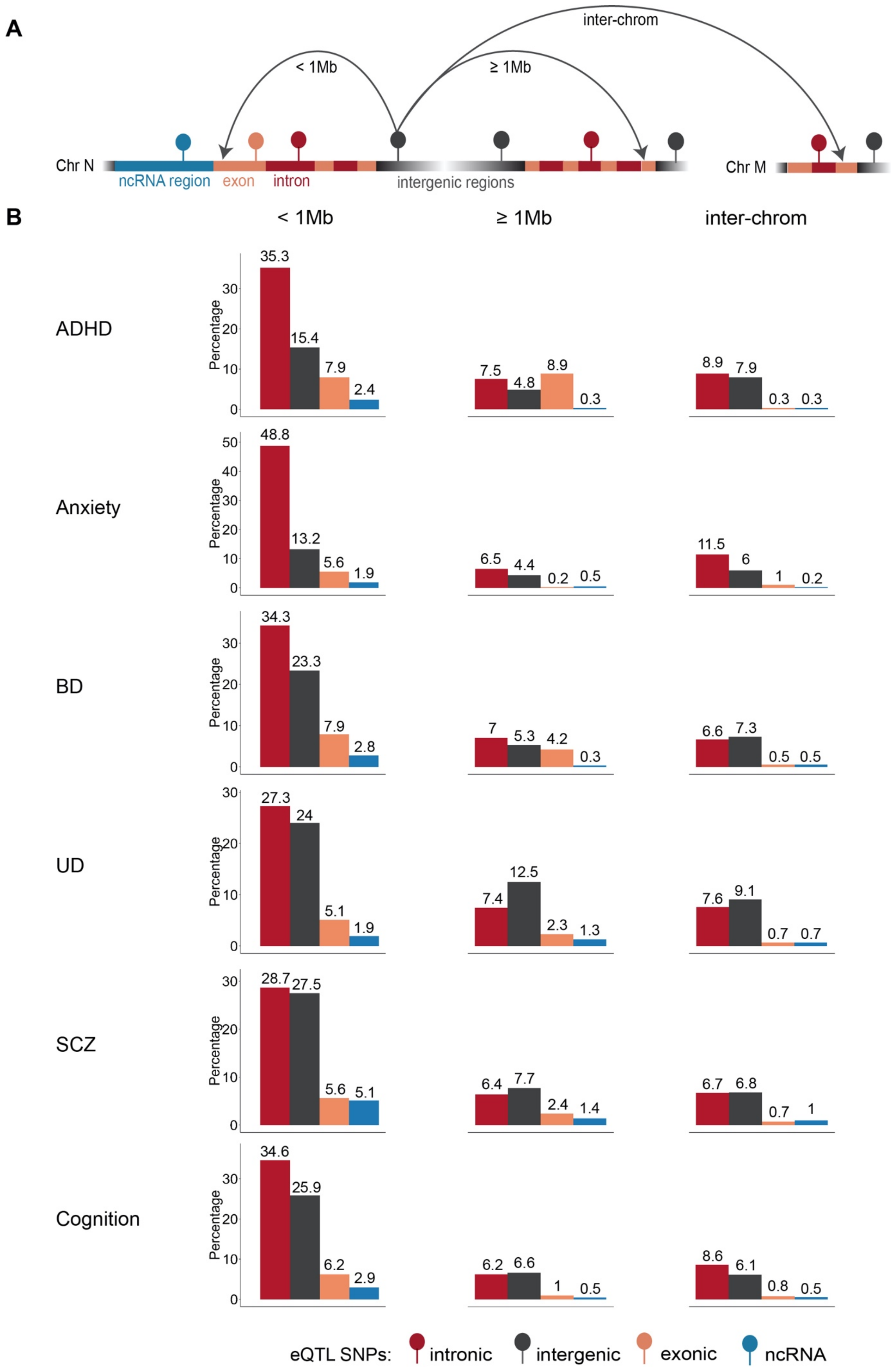

A
inter-chrom
< 1Mb
≥ 1Mb
Chr N
ncRNA region
exon
intron
intergenic regions
Chr M
B
< 1Mb
≥ 1Mb
inter-chrom
ADHD
Anxiety
BD
UD
SCZ
Cognition
Percentage
eQTL SNPs:
intronic
intergenic
exonic
ncRNA

Supplementary Figure 3. Functional types of eQTL SNPs associated with psychiatric disorders and cognition and the percentage distribution of these eQTL SNP types in the genome. **A** Schematic representation of cis- (< 1Mb) and trans-acting (≥ 1Mb and interchromosomal) SNPs. **B** Most of eQTL SNPs were enriched in intronic and intergenic regions and mark regions that regulate expression of genes in close proximity (i.e. in cis-manner). Functional annotation of trans-acting interactions showed that trans-eQTLs involve more coding SNPs (8.9%) in ADHD compared to other phenotypes. Trans-acting regulation in SCZ and UD is associated more with intergenic eQTLs than with intronic regulatory variants. The majority of interchromosomal eQTL effects are intronic and intergenic across all phenotypes.

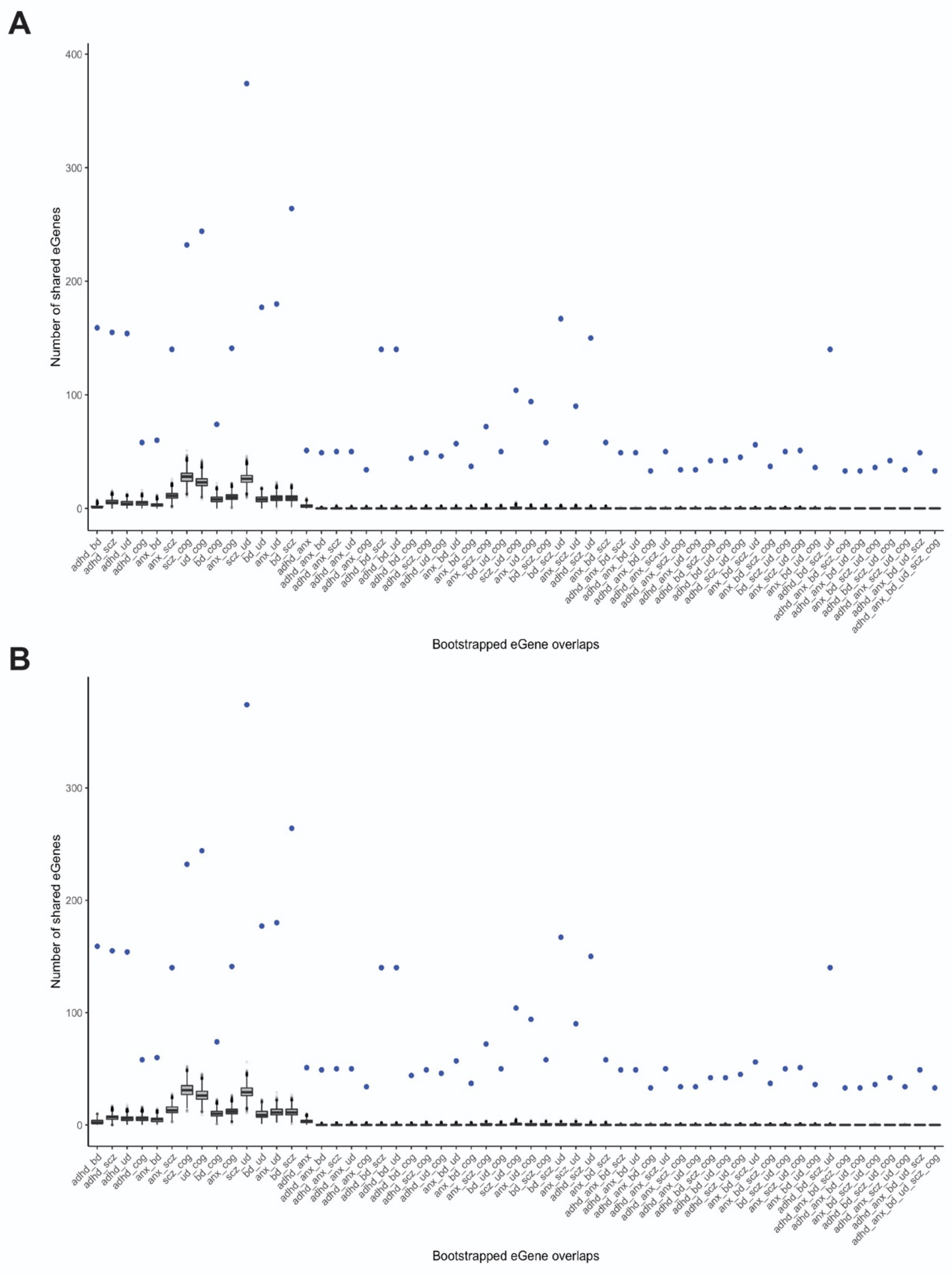

Supplementary Figure 4. Box plots of the bootstrapped (10,000 iterations) eGene overlaps among psychiatric disorders and cognition. All possible eGene overlaps amongst the five phenotypes are presented. **A** Bootstrap tests against all genes in the genome show that the observed eGene overlaps are statistically significant ($p < 0.001$). Blue dots indicate the observed number of shared eGenes from Figure 3C. For example, the observed eGene overlap across all five phenotypes (adhd_anx_bd_ud_scz_cog) is equal to 33 shared eGenes (Figure 3C). **B** Bootstrap tests against genes that were identified as interacting with the phenotype associated SNP containing fragments within the Hi-C libraries show that the observed eGene overlaps are statistically significant ($p < 0.001$). Blue dots indicate the observed number of shared eGenes from Figure 5B.

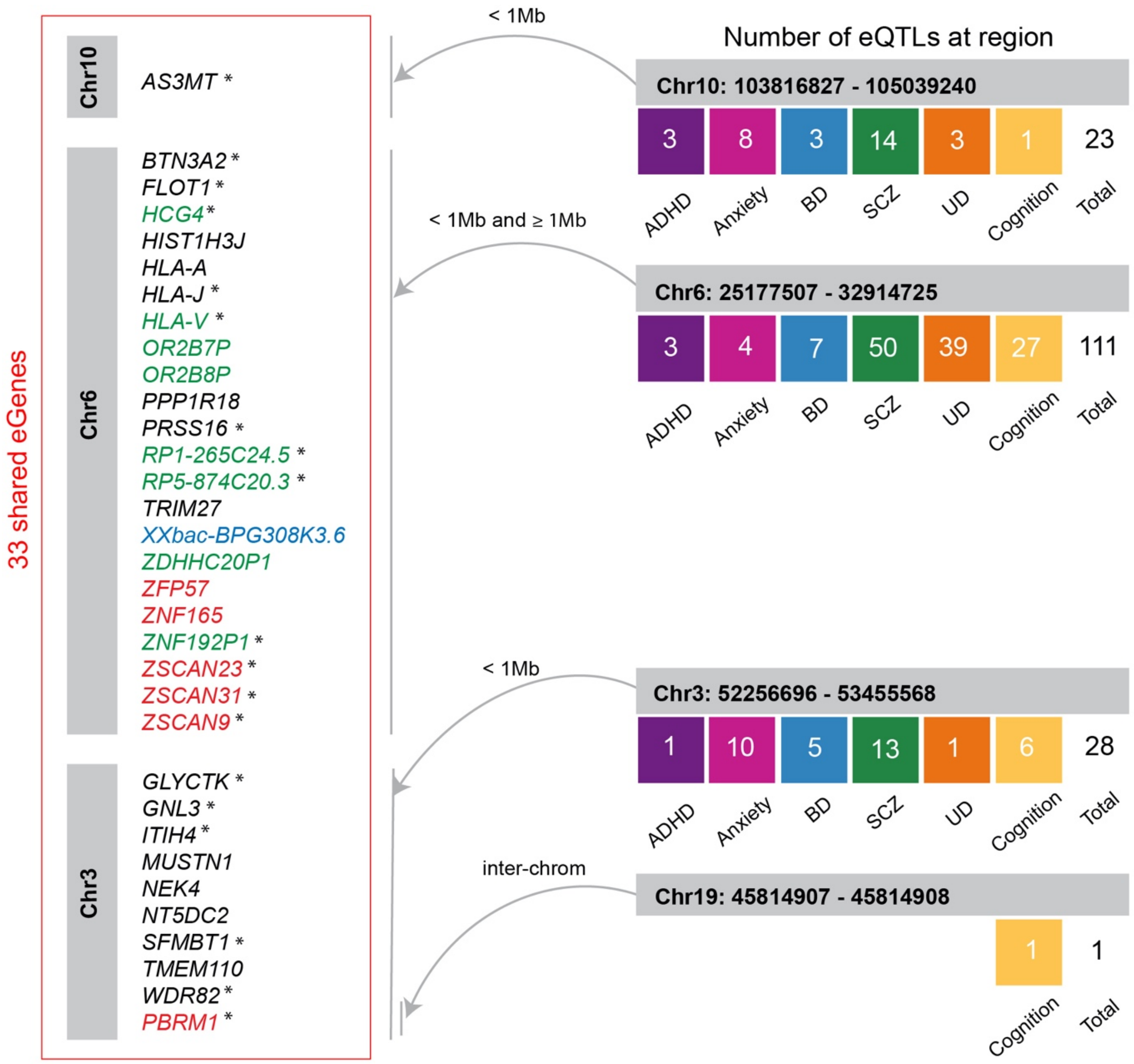

Supplementary Figure 5. Common eGenes are affected by different eQTLs. We identified thirty-three shared eGenes between psychiatric disorders and cognition. These are located on chromosomes 3, 6 and 10 and regulated in cis- and trans- by multiple eQTL SNPs from four putative regions on chromosomes 3, 6, 9 and 10. Most of the shared eGenes are protein-coding (colored in black). Six of them encode transcription factors (colored in red). eGenes colored in green indicate pseudogenes. One eGene (colored in blue) encodes ncRNA. eGenes marked with asterisks are associated with up- or downregulation in the brain.

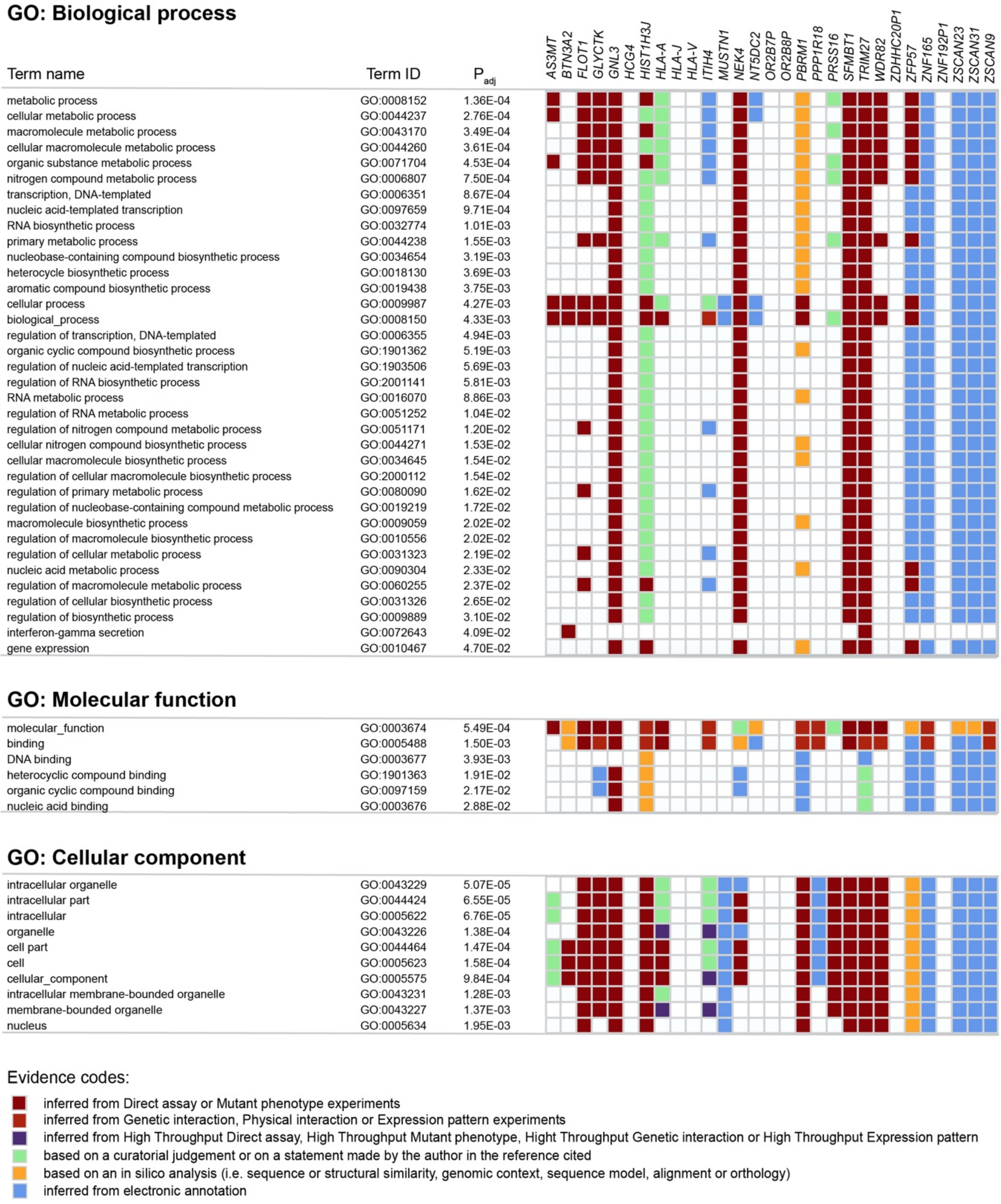

Supplementary Figure 6. Functional enrichment analysis for 33 shared eGenes (4 eGenes are not present in the g:GOSt database). Functional enrichment analysis for 33 shared eGenes (4 eGenes are not present in the g:GOSt database) showed that most of them are enriched in "binding" with DNA and other heterocyclic and organic cyclic compounds. Protein products of the shared eGenes are also involved in gene expression, transcription, interferon-gamma secretion and various metabolic, biosynthetic and regulatory processes within a cell. The adjusted $p < 0.05$ determines the significance level for GO terms.

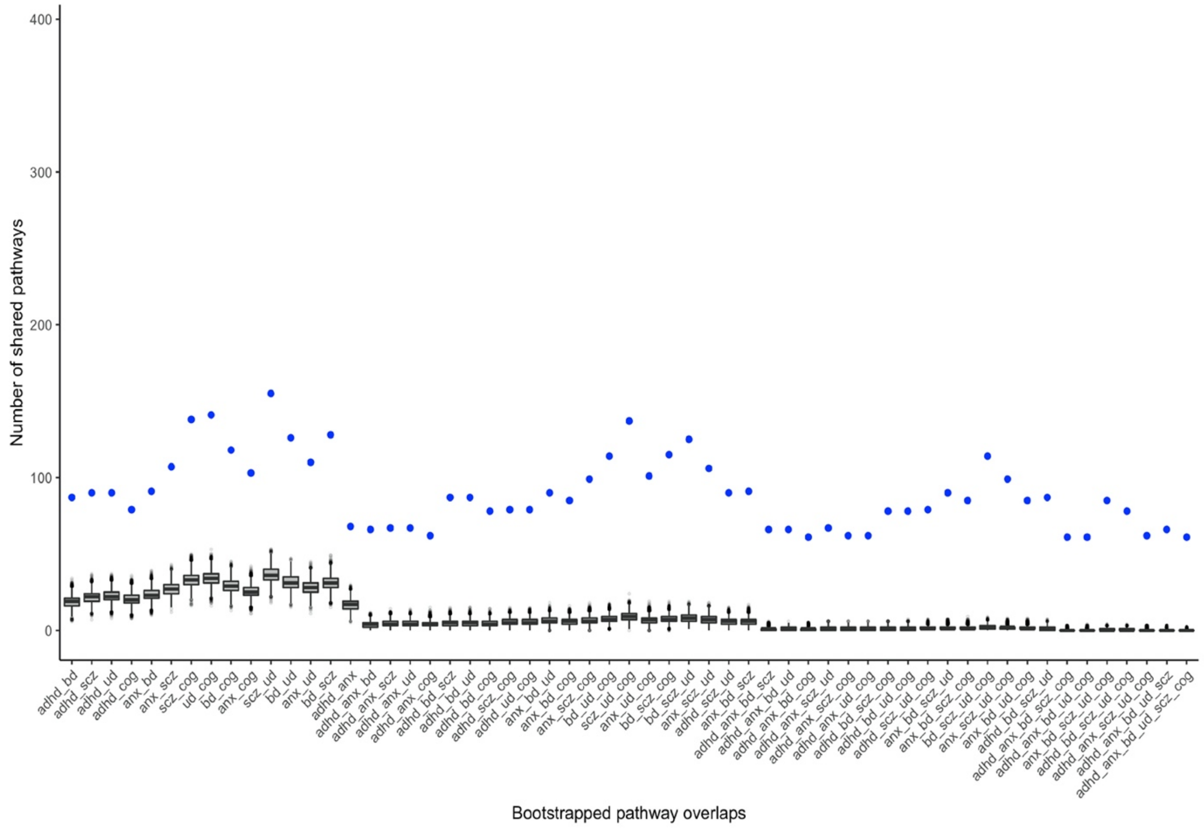

Supplementary Figure 7. Box plots of the bootstrapped (10,000 iterations) pathway overlaps among psychiatric disorders and cognition. All possible pathway overlaps amongst the five phenotypes are presented. The bootstrap test shows that the pathway overlaps are statistically significant ($p < 0.001$) and didn't occur due to chance. Blue dots indicate the number of observed shared pathways in Figure 3D. For example, the observed pathway overlap across all five phenotypes (adhd_anx_bd_ud_scz_cog) is equal to 61 shared pathways (Figure 3D).

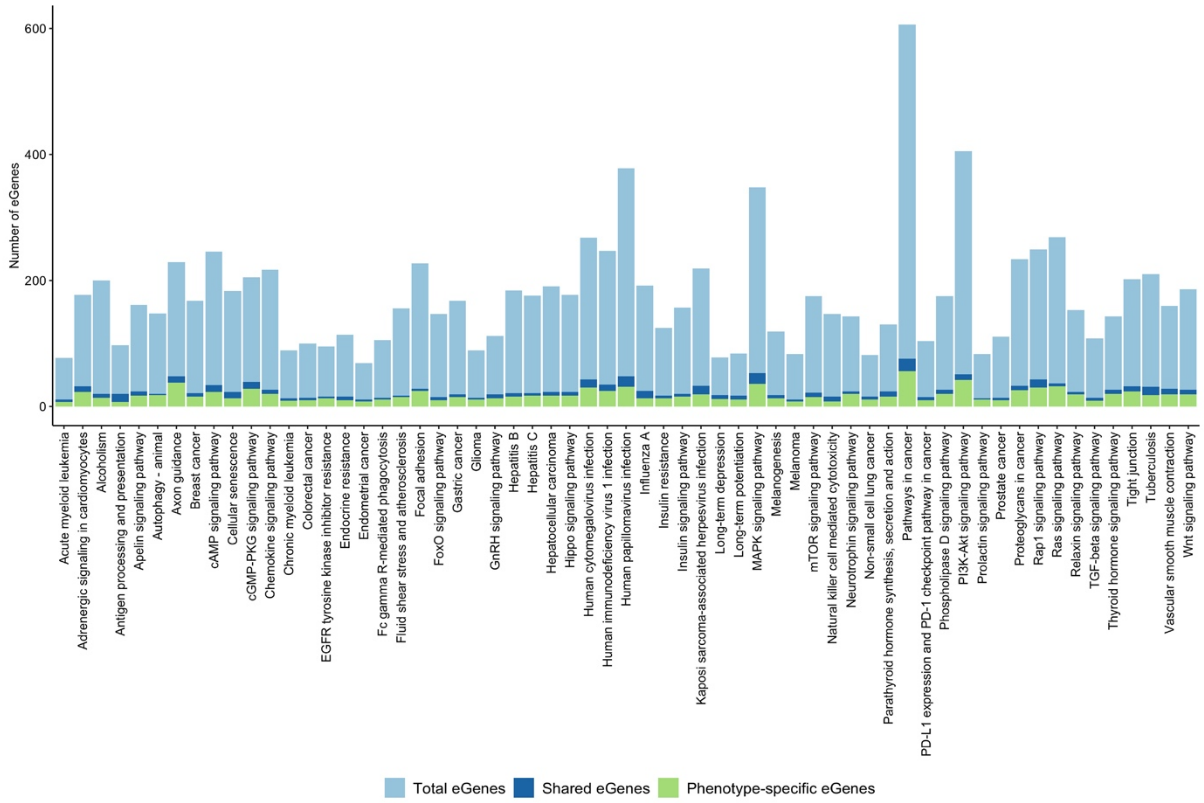

Supplementary Figure 8. Phenotype-specific eGenes and eGenes shared by at least two phenotypes are co-occurring within 61 shared biological pathways.

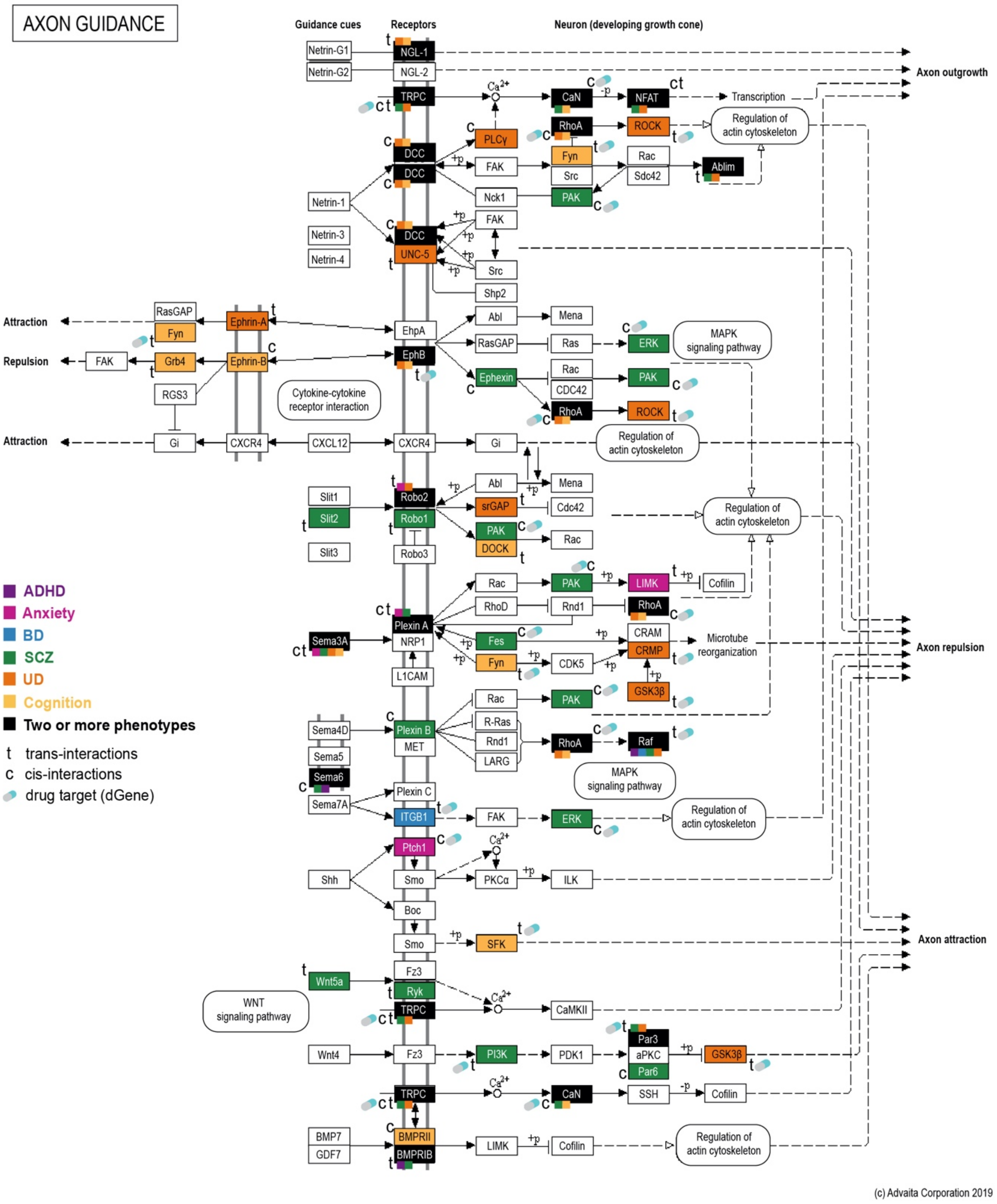

Supplementary Figure 9. SNPs mark eQTLs that act to regulate genes within the axon guidance pathway. Axon guidance represents a key stage in the development of neuronal networks. Axons are guided by netrins, ephrins, Slits, semaphorins and other guidance factors to reach their correct targets and form precise functional circuits. Co-occurrence of shared and phenotype-specific affected eGenes in this pathway may lead to a series of cellular events

associated with dysregulation and disintegration of these circuits in psychiatric and cognitive phenotypes.

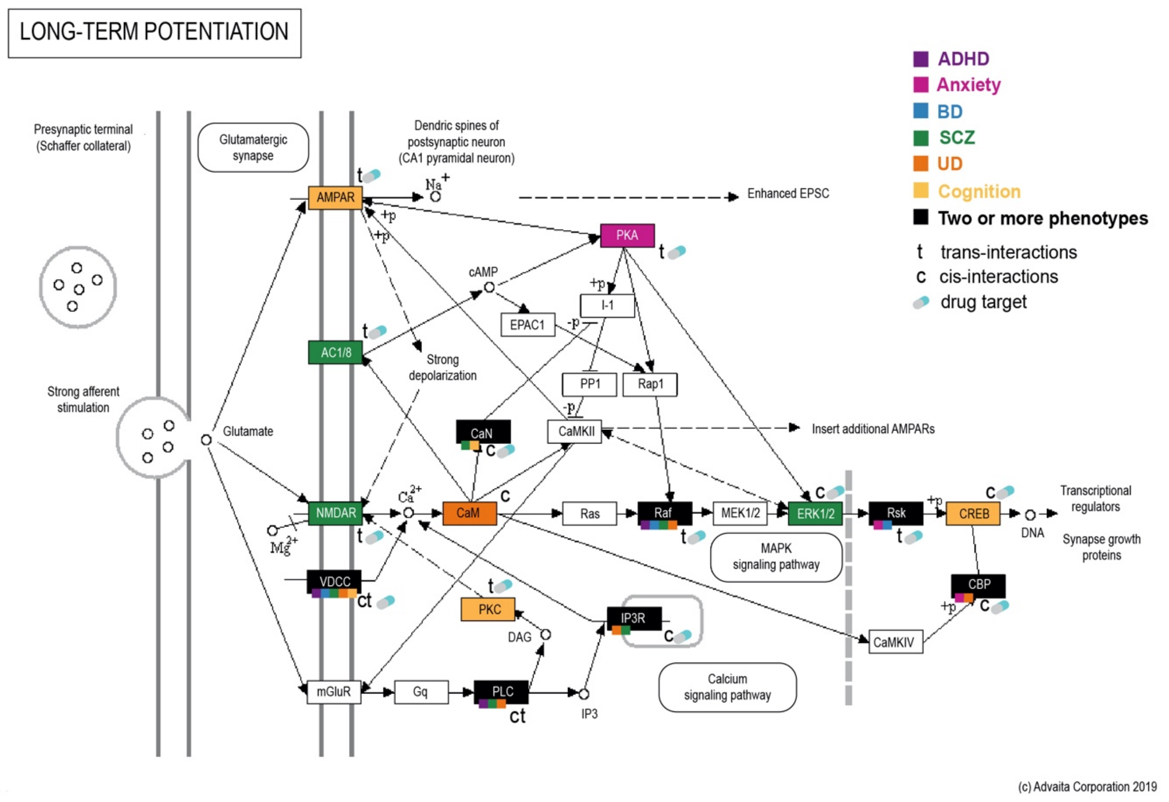

Supplementary Figure 10. Co-occurrence of genes regulated by phenotype-associated eQTLs within the long-term potentiation (LTP) pathway. LTP pathway - the molecular basis for learning and memory. Impaired LTP may have a role in psychiatric disorders and cognitive functioning.

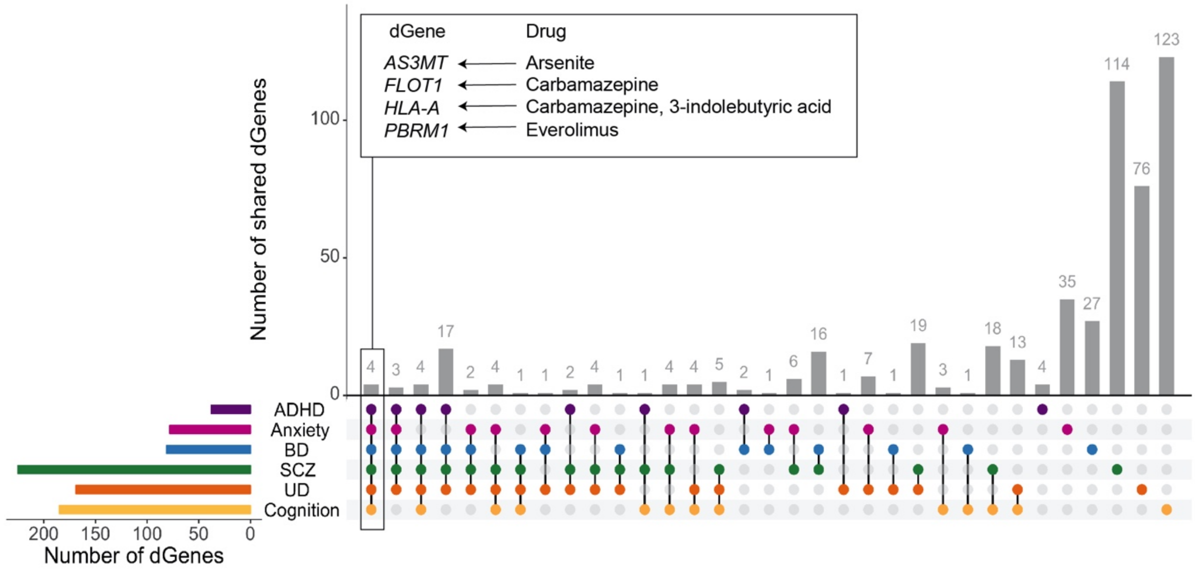

Supplementary Figure 11. Psychiatric disorders and cognition share druggable eGenes (dGenes) whose products represent potential drug targets. Four dGenes (*AS3MT*, *FLOT1*, *HLA-A* and *PBRM1*) are shared across all six phenotypes.

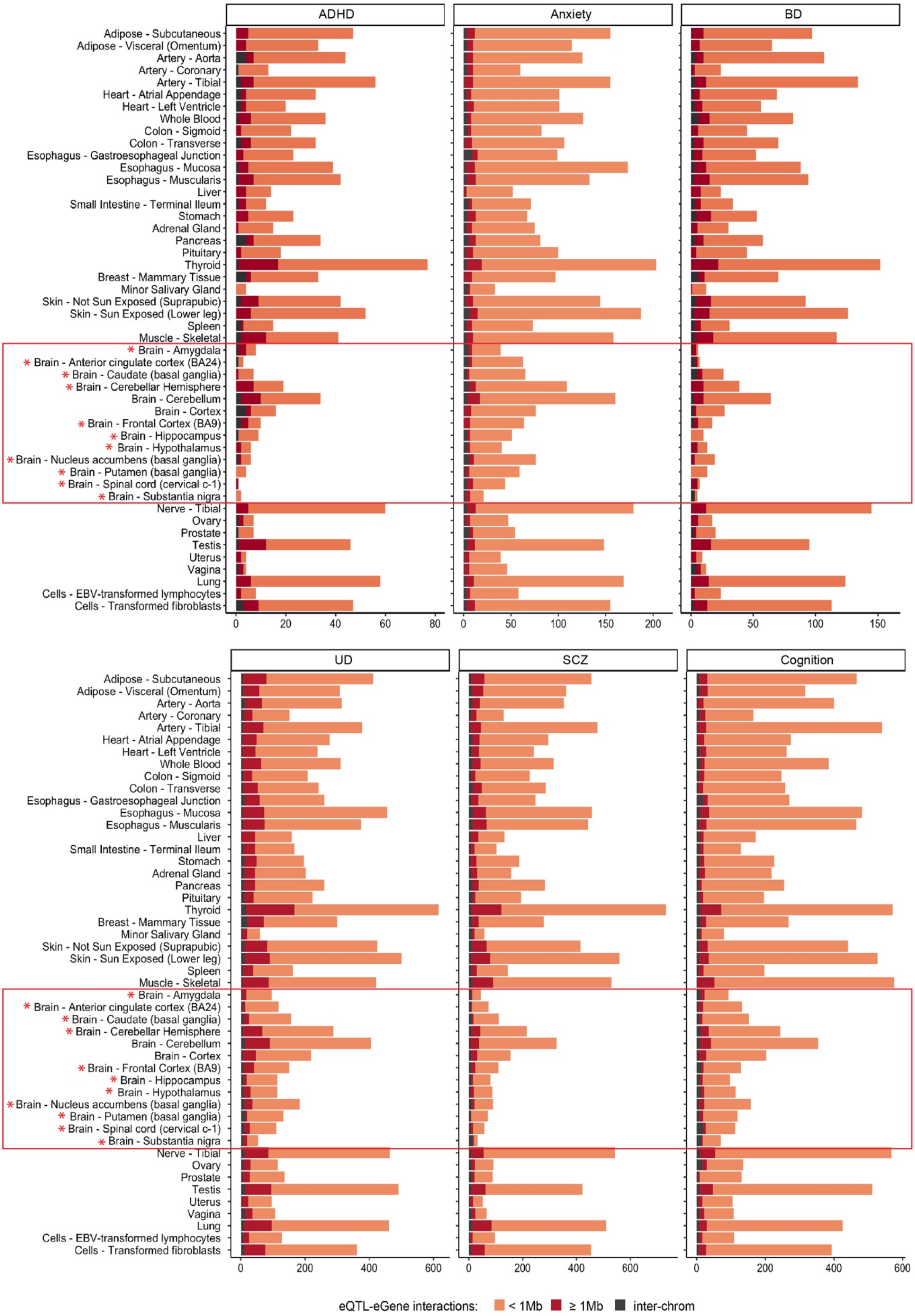

ADHD
Anxiety
BD
Adipose - Subcutaneous
Adipose - Visceral (Omentum)
Artery - Aorta
Artery - Coronary
Artery - Tibial
Heart - Atrial Appendage
Heart - Left Ventricle
Whole Blood
Colon - Sigmoid
Colon - Transverse
Esophagus - Gastroesophageal Junction
Esophagus - Mucosa
Esophagus - Muscularis
Liver
Small Intestine - Terminal Ileum
Stomach
Adrenal Gland
Pancreas
Pituitary
Thyroid
Breast - Mammary Tissue
Minor Salivary Gland
Skin - Not Sun Exposed (Suprapubic)
Skin - Sun Exposed (Lower leg)
Spleen
Muscle - Skeletal
* Brain - Amygdala
* Brain - Anterior cingulate cortex (BA24)
* Brain - Caudate (basal ganglia)
* Brain - Cerebellar Hemisphere
Brain - Cerebellum
Brain - Cortex
* Brain - Frontal Cortex (BA9)
* Brain - Hippocampus
* Brain - Hypothalamus
* Brain - Nucleus accumbens (basal ganglia)
* Brain - Putamen (basal ganglia)
* Brain - Spinal cord (cervical c-1)
* Brain - Substantia nigra
Nerve - Tibial
Ovary
Prostate
Testis
Uterus
Vagina
Lung
Cells - EBV-transformed lymphocytes
Cells - Transformed fibroblasts
0 20 40 60 80
0 50 100 150 200
0 50 100 150
UD
SCZ
Cognition
0 200 400 600
0 200 400 600
0 200 400 600
eQTL-eGene interactions: < 1Mb ≥ 1Mb inter-chrom

Supplementary Figure 12. Number of tissue-specific eQTL SNP-eGene interactions identified for psychiatric disorders and cognition. Red asterisks specify brain tissues that were re-sampled and have a longer ischemic time (the time from death until the time of the sample fixation) compared to other tissues.

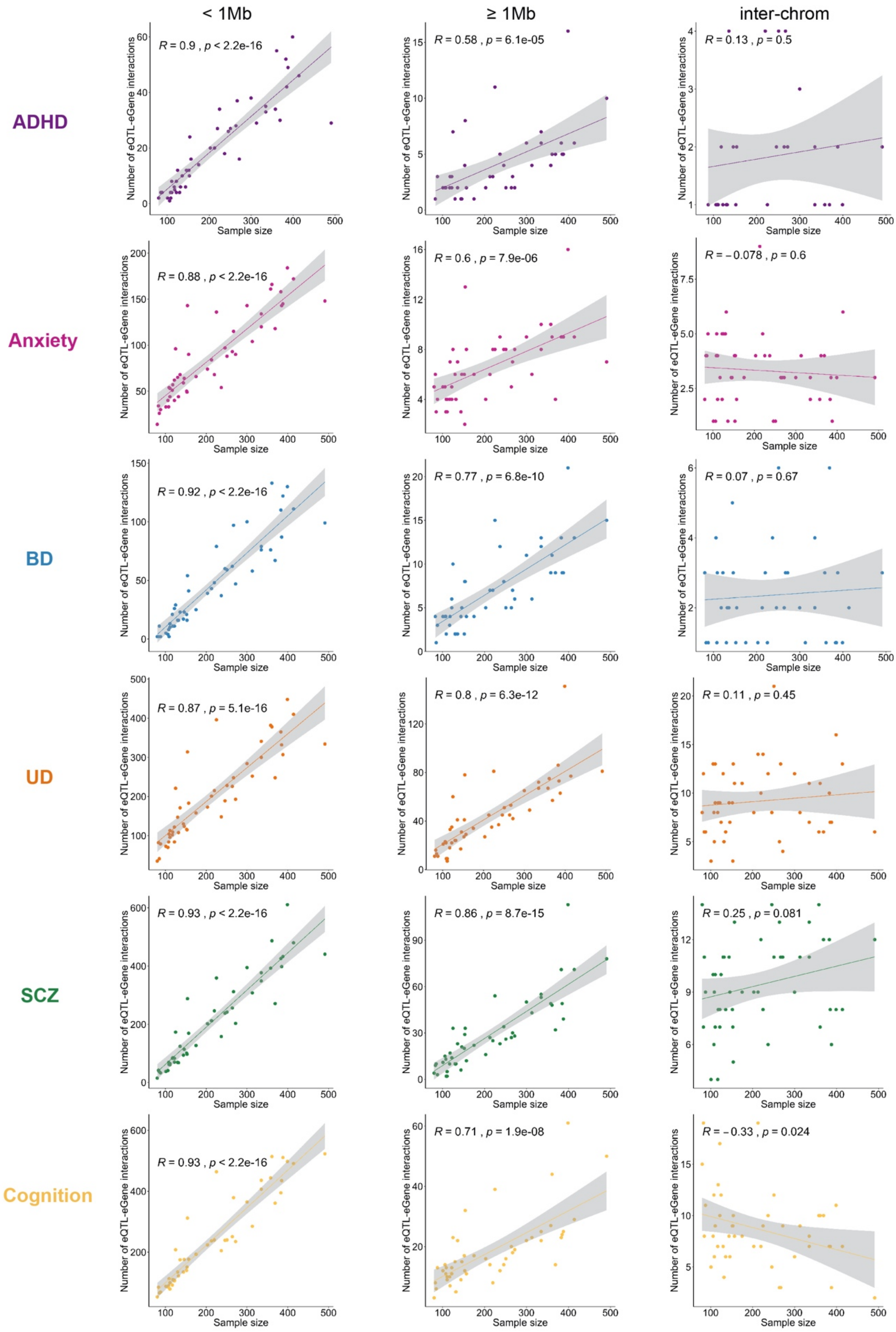

Supplementary Figure 13. Correlation analysis between GTEx tissue sample size and the number of cis- and trans-acting eQTL SNP-eGenes interactions identified for psychiatric disorders and cognition. Shaded regions represent 95% confidence intervals containing the true correlation. The larger is GTEx sample size, the more <1Mb and ≥1Mb eQTL SNP-

eGenes interactions are observed. However, the number of interchromosomal interactions tends to be not dependent on the GTEx sample size. For more information on cis- (<1Mb) and trans-acting (≥1Mb and interchromosomal) eQTL SNP-eGene interactions per GTEx tissue, see Supplementary Spreadsheet 6.

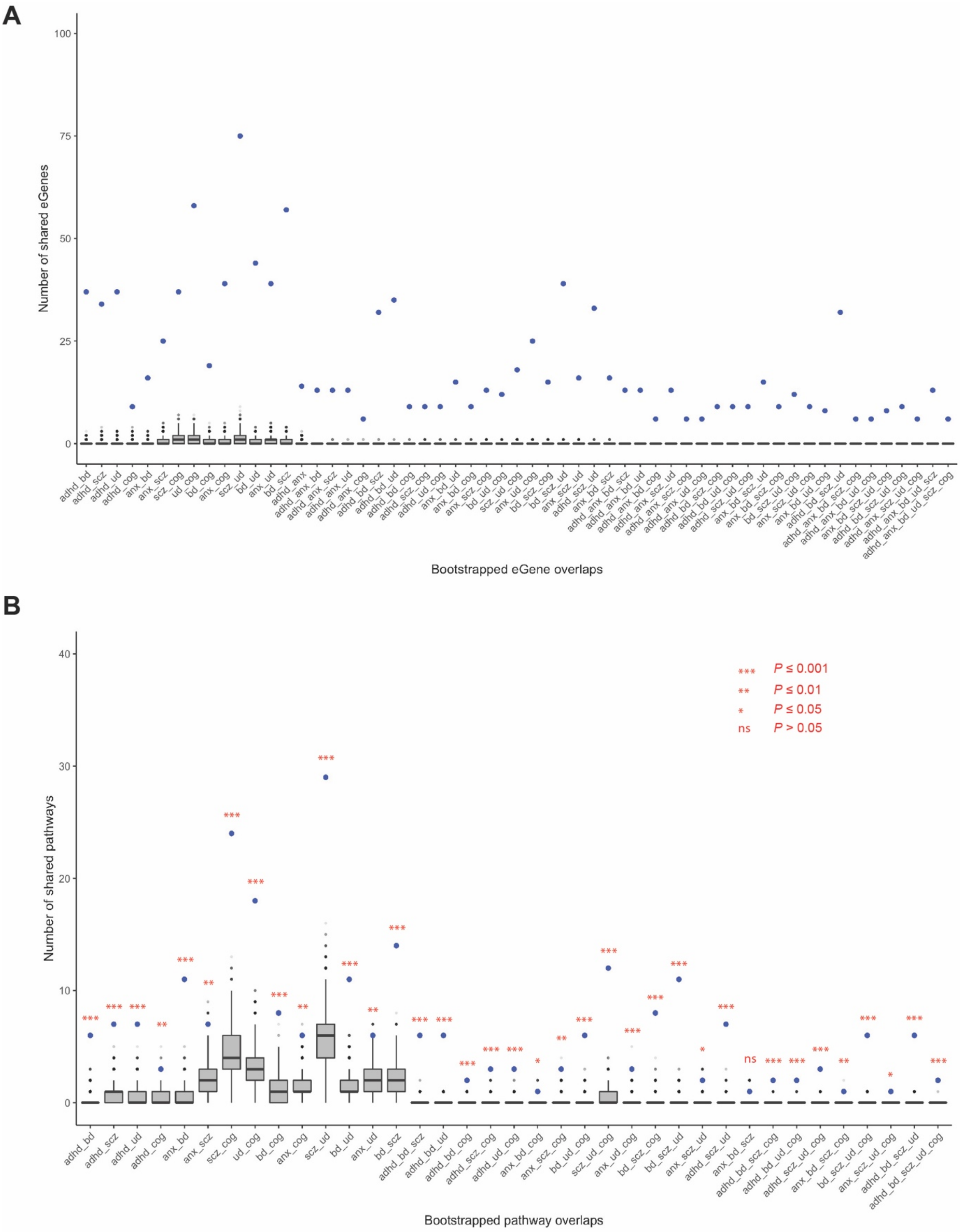

Supplementary Figure 14. Box plots of the bootstrapped (10,000 iterations) brain-related eGene and pathway overlaps among psychiatric disorders and cognition. Box plots of the bootstrapped (10,000 iterations) brain-related eGene (**A**) and pathway (**B**) overlaps among psychiatric disorders and cognition. Blue dots indicate the number of shared eGenes/pathways in the observed overlaps. The bootstrap test shows that brain-related eGenes overlaps are all statistically significant ($p < 0.001$) and didn’t occur due to chance. Most brain-related pathway overlaps are also statistically significant ($p < 0.05$).

3.2 Supplementary Tables

Supplementary Table 1. Hi-C datasets used in the analysis

№	Hi-C dataset	Number of replicates	Restriction enzyme	PubMed ID	GEO accession	Tissue
1	GM12878	23	MboI	25497547	GSE63525	peripheral blood, B cells
2	HMEC	6	MboI	25497547	GSE63525	breast, dermal endothelium
3	HUVEC	3	MboI	25497547	GSE63525	umbilical cord, umbilical vein
4	K562	6	MboI	25497547	GSE63525	blood, lymphoblasts
5	KBM7	5	MboI	25497547	GSE63525	blood, leukocytes
6	NHEK	3	MboI	25497547	GSE63525	skin, epidermis
7	IMR90	7	MboI	25497547	GSE63525	fetal lung
		2	HindIII	22495300	GSE35156	fetal lung
		6	HindIII	24141950	GSE43070	fetal lung
8	hESC	2	HindIII	22495300	GSE35156	embryo
		1	HindIII	24141950	GSE43070	embryo
9	Cortical plate neurons	3	HindIII	27760116	GSE77565	brain, cerebral cortex
10	Germinal plate neurons	3	HindIII	27760116	GSE77565	brain, cerebral cortex
11	Astrocyte of cerebellum	2	HindIII	22955616	GSE105194	brain, cerebellum
12	Brain vascular pericyte	1	HindIII	22955616	GSE105513	brain
13	Brain microvascular endothelial cell	2	HindIII	22955616	GSE105544	brain
14	Neuronal progenitor cells	2	HindIII	25693564	GSE52457	brain
15	SK-N-MC	2	HindIII	22955616	GSE105914	brain, supra-orbital area
16	Astrocyte of spinal cord	2	HindIII	22955616	GSE105957	spinal cord
17	HeLa	8	MboI	25497547	GSE63525	cervix
18	Adrenal gland	1	HindIII	27851967	GSE87112	adrenal gland cells

19	Bladder	2	HindIII	27851967	GSE87112	bladder cells
20	Dorsolateral prefrontal cortex	1	HindIII	27851967	GSE87112	brain, dorsolateral prefrontal cortex cells
21	Hippocampus	1	HindIII	27851967	GSE87112	brain, hippocampus cells
22	Lung	2	HindIII	27851967	GSE87112	lung cells
23	Ovary	1	HindIII	27851967	GSE87112	ovary cells
24	Pancreas	4	HindIII	27851967	GSE87112	pancreas, duct
25	Psoas	3	HindIII	27851967	GSE87112	psoas muscle cells
26	Right ventricle	1	HindIII	27851967	GSE87112	right ventricle cells
27	Small bowel	1	HindIII	27851967	GSE87112	small intestine cells
28	Spleen	2	HindIII	27851967	GSE87112	spleen cells

Supplementary Table 2. GTEx dataset v7 used in the analysis. GTEx v7 (dbGaP Accession phs000424.v7.p2) was used to analyse GWAS SNPs associated with ADHD, anxiety, BD, UD, SCZ and cognitive functioning.

№	GTEx tissue name	UBERON	Number of RNA-Seq and Genotyped samples	Number of RNA-Seq Samples	Number of eGenes
1	Adipose - Subcutaneous	2190	385	442	11866
2	Adipose - Visceral (Omentum)	10414	313	355	8991
3	Adrenal Gland	2369	175	190	6421
4	Artery - Aorta	1496	267	299	9205
5	Artery - Coronary	1621	152	173	4383
6	Artery - Tibial	7610	388	441	11947
7	Brain - Amygdala	1876	88	100	2229
8	Brain - Anterior cingulate cortex (BA24)	9835	109	121	4103
9	Brain - Caudate (basal ganglia)	1873	144	160	5700
10	Brain - Cerebellar Hemisphere	2037	125	136	6889
11	Brain - Cerebellum	2037	154	173	8429
12	Brain - Cortex	1870	136	158	6146

13	Brain - Frontal Cortex (BA9)	9834	118	129	5086
14	Brain - Hippocampus	1954	111	123	3262
15	Brain - Hypothalamus	1898	108	121	3077
16	Brain - Nucleus accumbens (basal ganglia)	1882	130	147	5118
17	Brain - Putamen (basal ganglia)	1874	111	124	4099
18	Brain - Spinal cord (cervical c-1)	6469	83	91	2644
19	Brain - Substantia nigra	2038	80	88	1807
20	Breast - Mammary Tissue	8367	251	290	6983
21	Cells - EBV-transformed lymphocytes	EFO_0000572	117	130	3845
22	Cells - Transformed fibroblasts	EFO_0002009	300	343	11091
23	Colon - Sigmoid	1159	203	233	7159
24	Colon - Transverse	1157	246	274	8094
25	Esophagus - Gastroesophageal Junction	4550	213	244	7254
26	Esophagus - Mucosa	6920	358	407	11694
27	Esophagus - Muscularis	4648	335	370	11071
28	Heart - Atrial Appendage	6631	264	297	8096
29	Heart - Left Ventricle	6566	272	303	7377
30	Liver	1114	153	175	4005
31	Lung	8952	383	427	11307
32	Minor Salivary Gland	6330	85	97	2150
33	Muscle - Skeletal	11907	491	564	10777
34	Nerve - Tibial	1323	361	414	13976
35	Ovary	992	122	133	3789
36	Pancreas	1150	220	248	7146
37	Pituitary	7	157	183	6263
38	Prostate	2367	132	152	4243
39	Skin - Not Sun Exposed (Suprapubic)	36149	335	387	10857
40	Skin - Sun Exposed (Lower leg)	4264	414	473	13109
41	Small Intestine - Terminal Ileum	1211	122	137	4763
42	Spleen	2106	146	162	6578
43	Stomach	945	237	262	6384

44	Testis	473	225	259	13759
45	Thyroid	2046	399	446	14313
46	Uterus	995	101	111	2641
47	Vagina	996	106	115	2527
48	Whole Blood	13756	369	407	8663

Supplementary Table 3. eGenes shared across all psychiatric disorders and cognitive functioning. Protein class information was obtained from the Human Protein Atlas (version 19.1; https://www.proteinatlas.org/).

№	Gene symbol	Gene ID	Gene type	Gene description	Protein class
1	*AS3MT*	ENSG00000214435.3	protein coding	Arsenite Methyltransferase	Enzymes<br>Predicted intracellular proteins
2	*BTN3A2*	ENSG00000186470.9	protein coding	Butyrophilin Subfamily 3 Member A2	Predicted intracellular proteins<br>Predicted membrane proteins<br>Predicted secreted proteins
3	*FLOT1*	ENSG00000137312.10	protein coding	Flotillin 1	Plasma proteins<br>Predicted intracellular proteins
4	*GLYCTK*	ENSG00000168237.13	protein coding	Glycerate Kinase	Disease related genes<br>Enzymes<br>Potential drug targets<br>Predicted intracellular proteins
5	*GNL3*	ENSG00000163938.12	protein coding	G Protein Nucleolar 3	Predicted intracellular proteins
6	*HCG4*	ENSG00000176998.3	pseudogene	HLA Complex Group 4 Pseudogene	-
7	*HIST1H3J*	ENSG00000197153.3	protein coding	Histone Cluster 1 H3 Family Member J	Predicted intracellular proteins
8	*HLA-A*	ENSG00000206503.7	protein coding	Major Histocompatibility Complex, Class I, A	Cancer-related genes<br>Plasma proteins<br>Predicted membrane proteins<br>Predicted secreted proteins
9	*HLA-J*	ENSG00000204622.6	protein coding	Major Histocompatibility Complex, Class I, C	Plasma proteins<br>Predicted membrane proteins<br>Predicted secreted proteins
10	*HLA-V*	ENSG00000181126.9	pseudogene	Major Histocompatibility Complex, Class I, V Pseudogene	-

11	*ITIH4*	ENSG00000055955.11	protein coding	Inter-Alpha-Trypsin Inhibitor Heavy Chain Family Member 4	Cancer-related genes<br>Plasma proteins<br>Predicted intracellular proteins<br>Predicted secreted proteins
12	*MUSTN1*	ENSG00000272573.1	protein coding	Musculoskeletal, Embryonic Nuclear Protein 1	Predicted intracellular proteins
13	*NEK4*	ENSG00000114904.8	protein coding	NIMA Related Kinase 4	Enzymes<br>Plasma proteins<br>Predicted intracellular proteins
14	*NT5DC2*	ENSG00000168268.6	protein coding	5'-Nucleotidase Domain Containing 2	Predicted intracellular proteins
15	*OR2B7P*	ENSG00000187763.3	pseudogene	Olfactory Receptor Family 2 Subfamily B Member 7 Pseudogene	-
16	*OR2B8P*	ENSG00000182477.5	pseudogene	Olfactory Receptor Family 2 Subfamily B Member 8 Pseudogene	-
17	*PBRM1*	ENSG00000163939.14	protein coding	Polybromo 1	Cancer-related genes<br>Disease related genes<br>Predicted intracellular proteins<br>Transcription factors
18	*PPP1R18*	ENSG00000146112.7	protein coding	Protein Phosphatase 1 Regulatory Subunit 18	Predicted intracellular proteins
19	*PRSS16*	ENSG00000112812.11	protein coding	Serine Protease 16	Enzymes<br>Predicted intracellular proteins<br>Predicted secreted proteins
20	*RP1-265C24.5*	ENSG00000219392.1	pseudogene		-
21	*RP5-874C20.3*	ENSG00000197062.7	pseudogene		-
22	*SFMBT1*	ENSG00000163935.9	protein coding	Scm Like With Four Mbt Domains 1	Predicted intracellular proteins
23	*TMEM110*	ENSG00000213533.7	protein coding	Transmembrane protein 110	Predicted intracellular proteins<br>Predicted membrane proteins<br>Transporters
24	*TRIM27*	ENSG00000204713.6	protein coding	Tripartite Motif Containing 27	Cancer-related genes<br>Disease related genes<br>Enzymes<br>Potential drug targets<br>Predicted intracellular proteins
25	*WDR82*	ENSG00000164091.7	protein coding	WD Repeat Domain 82	Predicted intracellular proteins

26	*XXbac-BPG308K3.6*	ENSG00000225595.2	ncRNA	Long Intergenic Non-Protein Coding RNA 1623	-
27	*ZDHHC20P1*	ENSG00000233916.1	pseudogene	Zinc Finger DHHC-Type Containing 20 Pseudogene 1	-
28	*ZFP57*	ENSG00000204644.5	protein coding	ZFP57 Zinc Finger Protein	Disease related genes<br>Predicted intracellular proteins<br>Transcription factors
29	*ZNF165*	ENSG00000197279.3	protein coding	Zinc Finger Protein 165	Predicted intracellular proteins<br>Transcription factors
30	*ZNF192P1*	ENSG00000226314.3	pseudogene	Zinc Finger Protein 192 Pseudogene 1	-
31	*ZSCAN23*	ENSG00000187987.5	protein coding	Zinc Finger And SCAN Domain Containing 23	Predicted intracellular proteins<br>Transcription factors
32	*ZSCAN31*	ENSG00000235109.3	protein coding	Zinc Finger And SCAN Domain Containing 31	Predicted intracellular proteins<br>Transcription factors
33	*ZSCAN9*	ENSG00000137185.7	protein coding	Zinc Finger And SCAN Domain Containing 9	Predicted intracellular proteins<br>Transcription factors

Supplementary Table 4. rs2535629 eQTL SNP-eGene associations linked to psychiatric disorders and cognitive functioning

eQTL SNP	eGene	Tissue	p-value	Adj_p-value	Effect_Size
ADHD					
rs2535629	*ITIH3*	Adipose_Subcutaneous	2.73E-10	3.46E-06	0.222223
rs2535629	*ITIH3*	Adipose_Visceral_Omentum	1.56E-06	0.006683630271	0.219241
rs2535629	*ITIH3*	Artery_Aorta	1.81E-19	1.35E-14	0.650073
rs2535629	*ITIH3*	Artery_Coronary	4.74E-09	4.55E-05	0.457006
rs2535629	*ITIH3*	Artery_Tibial	8.54E-20	7.04E-15	0.508774
rs2535629	*ITIH3*	Brain_Caudate_basal_ganglia	6.55E-06	0.0223210296	0.352338
rs2535629	*ITIH3*	Brain_Cerebellum	5.40E-07	0.002751683297	0.489369
rs2535629	*ITIH3*	Brain_Frontal_Cortex_BA9	1.08E-05	0.03331295367	0.416162
rs2535629	*ITIH3*	Brain_Hypothalamus	8.31E-07	0.003963562941	0.568006
rs2535629	*ITIH3*	Brain_Putamen_basal_ganglia	2.07E-06	0.008522065694	0.442893
rs2535629	*ITIH3*	Breast_Mammary_Tissue	1.63E-07	0.0009884034845	0.286252
rs2535629	*ITIH3*	Cells_EBV-transformed_lymphocytes	5.26E-07	0.002687800518	0.59652
rs2535629	*ITIH3*	Cells_Transformed_fibroblasts	1.14E-13	2.86E-09	0.444591
rs2535629	*ITIH3*	Colon_Transverse	7.14E-10	8.34E-06	0.249851
rs2535629	*ITIH3*	Esophagus_Mucosa	2.25E-12	4.23E-08	0.345726
rs2535629	*ITIH3*	Esophagus_Muscularis	4.90E-07	0.002541383362	0.188342
rs2535629	*ITIH3*	Heart_Atrial_Appendage	3.52E-11	5.29E-07	0.429801

rs2535629	*ITIH3*	Lung	4.96E-17	2.21E-12	0.401567
rs2535629	*ITIH3*	Muscle_Skeletal	9.48E-11	1.30E-06	0.182986
rs2535629	*ITIH3*	Nerve_Tibial	1.87E-12	3.59E-08	0.294419
rs2535629	*ITIH3*	Pituitary	2.84E-07	0.001568810916	0.518171
rs2535629	*ITIH3*	Skin_Not_Sun_Exposed_Suprapubic	1.33E-11	2.17E-07	0.310305
rs2535629	*ITIH3*	Skin_Sun_Exposed_Lower_leg	6.05E-14	1.60E-09	0.273844
rs2535629	*ITIH3*	Small_Intestine_Terminal_Ileum	4.99E-06	0.01787987193	0.294474
rs2535629	*ITIH3*	Thyroid	1.30E-19	9.99E-15	0.387306
rs2535629	*ITIH3*	Whole_Blood	4.27E-18	2.31E-13	0.340851
rs2535629	*NEK4*	Adipose_Visceral_Omentum	8.62E-06	0.02776253443	-0.187477
rs2535629	*NEK4*	Artery_Tibial	2.47E-06	0.009879884126	-0.185873
rs2535629	*NEK4*	Breast_Mammary_Tissue	8.21E-07	0.00393969213	-0.261104
rs2535629	*NEK4*	Cells_Transformed_fibroblasts	8.24E-07	0.00394537091	-0.201519
rs2535629	*NEK4*	Esophagus_Mucosa	1.95E-06	0.008099786356	-0.220788
rs2535629	*NEK4*	Heart_Atrial_Appendage	5.63E-07	0.002851329833	-0.245985
rs2535629	*NEK4*	Minor_Salivary_Gland	3.77E-06	0.01392594723	-0.476938
rs2535629	*NEK4*	Nerve_Tibial	1.22E-08	0.0001040712726	-0.23281
rs2535629	*NEK4*	Skin_Sun_Exposed_Lower_leg	1.65E-08	0.0001361613472	-0.185539
rs2535629	*NEK4*	Thyroid	2.06E-07	0.001197323228	-0.196945
rs2535629	*MUSTN1*	Artery_Aorta	5.16E-13	1.10E-08	0.487663
rs2535629	*MUSTN1*	Whole_Blood	1.03E-06	0.004776037304	0.189607
rs2535629	*TMEM110*	Artery_Aorta	9.69E-09	8.56E-05	0.317929
rs2535629	*TMEM110*	Lung	1.45E-05	0.04228619937	0.184534
rs2535629	*TMEM110*	Muscle_Skeletal	1.11E-09	1.22E-05	0.20707
rs2535629	*TMEM110*	Nerve_Tibial	1.47E-06	0.006345194956	0.197601
rs2535629	*TMEM110*	Whole_Blood	7.45E-06	0.02457292285	0.121372
rs2535629	*SFMBT1*	Esophagus_Gastroesophageal_Junction	1.57E-05	0.04482971953	0.245853
rs2535629	*SFMBT1*	Whole_Blood	8.72E-06	0.02799613658	0.147396
rs2535629	*GNL3*	Artery_Coronary	3.43E-06	0.01290831383	-0.174479
rs2535629	*GNL3*	Artery_Tibial	2.40E-07	0.001368306863	-0.161703
rs2535629	*GNL3*	Brain_Cerebellum	4.82E-08	0.0003445943968	-0.447907
rs2535629	*GNL3*	Cells_Transformed_fibroblasts	1.64E-08	0.0001361613472	-0.126615
rs2535629	*GNL3*	Esophagus_Muscularis	1.33E-06	0.005845357753	-0.171801
rs2535629	*GNL3*	Heart_Left_Ventricle	2.34E-07	0.001336454872	-0.137947
rs2535629	*GNL3*	Nerve_Tibial	2.15E-07	0.001240939774	-0.126861
rs2535629	*GNL3*	Pituitary	3.46E-07	0.001892086251	-0.223075
rs2535629	*GNL3*	Testis	1.51E-07	0.0009211473899	-0.204794
rs2535629	*PBRM1*	Artery_Tibial	3.67E-13	8.11E-09	0.158823
rs2535629	*PBRM1*	Cells_Transformed_fibroblasts	7.30E-06	0.02421558501	-0.113194
rs2535629	*PBRM1*	Thyroid	4.25E-29	1.08E-23	0.371498
rs2535629	*NT5DC2*	Cells_EBV-transformed_lymphocytes	2.45E-06	0.009782529373	-0.40891
rs2535629	*NT5DC2*	Cells_Transformed_fibroblasts	2.45E-07	0.001394208722	-0.104395
rs2535629	*NT5DC2*	Colon_Transverse	4.54E-08	0.0003271098835	-0.249637
rs2535629	*NT5DC2*	Skin_Not_Sun_Exposed_Suprapubic	4.91E-09	4.69E-05	-0.188731

rs2535629	*NT5DC2*	Skin_Sun_Exposed_Lower_leg	2.51E-10	3.21E-06	-0.192675
rs2535629	*NT5DC2*	Thyroid	6.80E-11	9.56E-07	-0.226272
rs2535629	*GLYCTK*	Lung	1.93E-07	0.001139289292	-0.175762
rs2535629	*GLYCTK*	Nerve_Tibial	2.18E-06	0.008853394276	-0.20308
rs2535629	*WDR82*	Cells_Transformed_fibroblasts	7.89E-06	0.02573057123	0.134094
rs2535629	*PPM1M*	Brain_Cerebellar_Hemisphere	6.56E-06	0.02232406944	-0.357333
rs2535629	*PPM1M*	Brain_Cerebellum	6.98E-07	0.003423762298	-0.396815
rs2535629	*RAF1*	Cells_EBV-transformed_lymphocytes	6.26E-06	0.02152655312	-0.3826631664
**Bipolar disorder**					
rs2535629	*ITIH4*	Adipose_Subcutaneous	2.73E-10	3.71E-06	0.222223
rs2535629	*ITIH4*	Adipose_Visceral_Omentum	1.56E-06	0.007671234989	0.219241
rs2535629	*ITIH4*	Artery_Aorta	1.81E-19	1.54E-14	0.650073
rs2535629	*ITIH4*	Artery_Coronary	4.74E-09	4.75E-05	0.457006
rs2535629	*ITIH4*	Artery_Tibial	8.54E-20	7.78E-15	0.508774
rs2535629	*ITIH4*	Brain_Caudate_basal_ganglia	6.55E-06	0.02542890743	0.352338
rs2535629	*ITIH4*	Brain_Cerebellum	5.40E-07	0.003133509221	0.489369
rs2535629	*ITIH4*	Brain_Frontal_Cortex_BA9	1.08E-05	0.0380487843	0.416162
rs2535629	*ITIH4*	Brain_Hypothalamus	8.31E-07	0.004492308226	0.568006
rs2535629	*ITIH4*	Brain_Putamen_basal_ganglia	2.07E-06	0.009791604266	0.442893
rs2535629	*ITIH4*	Breast_Mammary_Tissue	1.63E-07	0.00109742519	0.286252
rs2535629	*ITIH4*	Cells_EBV-transformed_lymphocytes	5.26E-07	0.003059503036	0.59652
rs2535629	*ITIH4*	Cells_Transformed_fibroblasts	1.14E-13	3.26E-09	0.444591
rs2535629	*ITIH4*	Colon_Transverse	7.14E-10	8.80E-06	0.249851
rs2535629	*ITIH4*	Esophagus_Mucosa	2.25E-12	4.78E-08	0.345726
rs2535629	*ITIH4*	Esophagus_Muscularis	4.90E-07	0.002881519899	0.188342
rs2535629	*ITIH4*	Heart_Atrial_Appendage	3.52E-11	5.74E-07	0.429801
rs2535629	*ITIH4*	Lung	4.96E-17	2.56E-12	0.401567
rs2535629	*ITIH4*	Muscle_Skeletal	9.48E-11	1.42E-06	0.182986
rs2535629	*ITIH4*	Nerve_Tibial	1.87E-12	4.05E-08	0.294419
rs2535629	*ITIH4*	Pituitary	2.84E-07	0.001765508363	0.518171
rs2535629	*ITIH4*	Skin_Not_Sun_Exposed_Suprapubic	1.33E-11	2.40E-07	0.310305
rs2535629	*ITIH4*	Skin_Sun_Exposed_Lower_leg	6.05E-14	1.79E-09	0.273844
rs2535629	*ITIH4*	Small_Intestine_Terminal_Ileum	4.99E-06	0.02044324371	0.294474
rs2535629	*ITIH4*	Thyroid	1.30E-19	1.14E-14	0.387306
rs2535629	*ITIH4*	Whole_Blood	4.27E-18	2.87E-13	0.340851
rs2535629	*NEK4*	Adipose_Visceral_Omentum	8.62E-06	0.03144250445	-0.187477
rs2535629	*NEK4*	Artery_Tibial	2.47E-06	0.01137232259	-0.185873
rs2535629	*NEK4*	Breast_Mammary_Tissue	8.21E-07	0.004453312859	-0.261104
rs2535629	*NEK4*	Cells_Transformed_fibroblasts	8.24E-07	0.004463426957	-0.201519
rs2535629	*NEK4*	Esophagus_Mucosa	1.95E-06	0.009316679055	-0.220788
rs2535629	*NEK4*	Heart_Atrial_Appendage	5.63E-07	0.003251800441	-0.245985
rs2535629	*NEK4*	Minor_Salivary_Gland	3.77E-06	0.01607915984	-0.476938
rs2535629	*NEK4*	Nerve_Tibial	1.22E-08	0.0001093283523	-0.23281
rs2535629	*NEK4*	Skin_Sun_Exposed_Lower_leg	1.65E-08	0.0001448396867	-0.185539

rs2535629	*NEK4*	Thyroid	2.06E-07	0.001332402457	-0.196945
rs2535629	*MUSTN1*	Artery_Aorta	5.16E-13	1.27E-08	0.487663
rs2535629	*MUSTN1*	Whole_Blood	1.03E-06	0.00542746235	0.189607
rs2535629	*TMEM110*	Artery_Aorta	9.69E-09	8.87E-05	0.317929
rs2535629	*TMEM110*	Lung	1.45E-05	0.04791223921	0.184534
rs2535629	*TMEM110*	Muscle_Skeletal	1.11E-09	1.28E-05	0.20707
rs2535629	*TMEM110*	Nerve_Tibial	1.47E-06	0.007271459026	0.197601
rs2535629	*TMEM110*	Whole_Blood	7.45E-06	0.02801578259	0.121372
rs2535629	*SFMBT1*	Whole_Blood	8.72E-06	0.03174651812	0.147396
rs2535629	*GNL3*	Artery_Coronary	3.43E-06	0.01495373915	-0.174479
rs2535629	*GNL3*	Artery_Tibial	2.40E-07	0.001528230837	-0.161703
rs2535629	*GNL3*	Brain_Cerebellum	4.82E-08	0.0003733289529	-0.447907
rs2535629	*GNL3*	Cells_Transformed_fibroblasts	1.64E-08	0.0001447936683	-0.126615
rs2535629	*GNL3*	Esophagus_Muscularis	1.33E-06	0.006678936492	-0.171801
rs2535629	*GNL3*	Heart_Left_Ventricle	2.34E-07	0.001491170878	-0.137947
rs2535629	*GNL3*	Nerve_Tibial	2.15E-07	0.00138175758	-0.126861
rs2535629	*GNL3*	Pituitary	3.46E-07	0.002110885335	-0.223075
rs2535629	*GNL3*	Testis	1.51E-07	0.001025073343	-0.204794
rs2535629	*PBRM1*	Artery_Tibial	3.67E-13	9.40E-09	0.158823
rs2535629	*PBRM1*	Cells_Transformed_fibroblasts	7.30E-06	0.02753830661	-0.113194
rs2535629	*PBRM1*	Thyroid	4.25E-29	1.41E-23	0.371498
rs2535629	*NT5DC2*	Cells_EBV-transformed_lymphocytes	2.45E-06	0.01127238706	-0.40891
rs2535629	*NT5DC2*	Cells_Transformed_fibroblasts	2.45E-07	0.001557962517	-0.104395
rs2535629	*NT5DC2*	Colon_Transverse	4.54E-08	0.0003536583851	-0.249637
rs2535629	*NT5DC2*	Skin_Not_Sun_Exposed_Suprapubic	4.91E-09	4.90E-05	-0.188731
rs2535629	*NT5DC2*	Skin_Sun_Exposed_Lower_leg	2.51E-10	3.43E-06	-0.192675
rs2535629	*NT5DC2*	Thyroid	6.80E-11	1.04E-06	-0.226272
rs2535629	*GLYCTK*	Lung	1.93E-07	0.00126661979	-0.175762
rs2535629	*GLYCTK*	Nerve_Tibial	2.18E-06	0.01022508236	-0.20308
rs2535629	*WDR82*	Cells_Transformed_fibroblasts	7.89E-06	0.02926604996	0.134094
rs2535629	*PPM1M*	Brain_Cerebellar_Hemisphere	6.56E-06	0.02546212486	-0.357333
rs2535629	*PPM1M*	Brain_Cerebellum	6.98E-07	0.003887934463	-0.396815
rs2535629	*RAF1*	Cells_EBV-transformed_lymphocytes	6.26E-06	0.0245266372	-0.3826631664
**Unipolar depression**					
rs2535629	*ITIH4*	Adipose_Subcutaneous	2.73E-10	1.77E-06	0.222223
rs2535629	*ITIH4*	Adipose_Visceral_Omentum	1.56E-06	0.004963531035	0.219241
rs2535629	*ITIH4*	Artery_Aorta	1.81E-19	3.36E-15	0.650073
rs2535629	*ITIH4*	Artery_Coronary	4.74E-09	2.53E-05	0.457006
rs2535629	*ITIH4*	Artery_Tibial	8.54E-20	1.64E-15	0.508774
rs2535629	*ITIH4*	Brain_Caudate_basal_ganglia	6.55E-06	0.01755078458	0.352338
rs2535629	*ITIH4*	Brain_Cerebellum	5.40E-07	1.93E-03	0.489369
rs2535629	*ITIH4*	Brain_Cortex	2.07E-05	0.04691722813	0.441604
rs2535629	*ITIH4*	Brain_Frontal_Cortex_BA9	1.08E-05	0.02714353417	0.416162
rs2535629	*ITIH4*	Brain_Hypothalamus	8.31E-07	0.002829190964	0.568006

rs2535629	*ITIH4*	Brain_Putamen_basal_ganglia	2.07E-06	0.00636171129	0.442893
rs2535629	*ITIH4*	Breast_Mammary_Tissue	1.63E-07	0.0006536217047	0.286252
rs2535629	*ITIH4*	Cells_EBV-transformed_lymphocytes	5.26E-07	0.001878732941	0.59652
rs2535629	*ITIH4*	Cells_Transformed_fibroblasts	1.14E-13	1.13E-09	0.444591
rs2535629	*ITIH4*	Colon_Transverse	7.14E-10	4.34E-06	0.249851
rs2535629	*ITIH4*	Esophagus_Mucosa	2.25E-12	1.93E-08	0.345726
rs2535629	*ITIH4*	Esophagus_Muscularis	4.90E-07	1.76E-03	0.188342
rs2535629	*ITIH4*	Heart_Atrial_Appendage	3.52E-11	2.57E-07	0.429801
rs2535629	*ITIH4*	Lung	4.96E-17	7.20E-13	0.401567
rs2535629	*ITIH4*	Muscle_Skeletal	9.48E-11	6.53E-07	0.182986
rs2535629	*ITIH4*	Nerve_Tibial	1.87E-12	1.63E-08	0.294419
rs2535629	*ITIH4*	Pituitary	2.84E-07	1.07E-03	0.518171
rs2535629	*ITIH4*	Skin_Not_Sun_Exposed_Suprapubic	1.33E-11	1.03E-07	0.310305
rs2535629	*ITIH4*	Skin_Sun_Exposed_Lower_leg	6.05E-14	6.22E-10	0.273844
rs2535629	*ITIH4*	Small_Intestine_Terminal_Ileum	4.99E-06	0.01382434431	0.294474
rs2535629	*ITIH4*	Thyroid	1.30E-19	2.45E-15	0.387306
rs2535629	*ITIH4*	Whole_Blood	4.27E-18	6.99E-14	0.340851
rs2535629	*NEK4*	Adipose_Visceral_Omentum	8.62E-06	0.02226688486	-0.187477
rs2535629	*NEK4*	Artery_Tibial	2.47E-06	7.45E-03	-0.185873
rs2535629	*NEK4*	Breast_Mammary_Tissue	8.21E-07	0.00279904157	-0.261104
rs2535629	*NEK4*	Cells_Transformed_fibroblasts	8.24E-07	0.002806096384	-0.201519
rs2535629	*NEK4*	Esophagus_Mucosa	1.95E-06	6.04E-03	-0.220788
rs2535629	*NEK4*	Heart_Atrial_Appendage	5.63E-07	2.00E-03	-0.245985
rs2535629	*NEK4*	Minor_Salivary_Gland	3.77E-06	0.01082222205	-0.476938
rs2535629	*NEK4*	Nerve_Tibial	1.22E-08	6.08E-05	-0.23281
rs2535629	*NEK4*	Skin_Sun_Exposed_Lower_leg	1.65E-08	8.01E-05	-0.185539
rs2535629	*NEK4*	Thyroid	2.06E-07	8.07E-04	-0.196945
rs2535629	*MUSTN1*	Artery_Aorta	5.16E-13	4.77E-09	0.487663
rs2535629	*MUSTN1*	Whole_Blood	1.03E-06	0.003432919765	0.189607
rs2535629	*TMEM110*	Artery_Aorta	9.69E-09	4.92E-05	0.317929
rs2535629	*TMEM110*	Lung	1.45E-05	3.48E-02	0.184534
rs2535629	*TMEM110*	Muscle_Skeletal	1.11E-09	6.53E-06	0.20707
rs2535629	*TMEM110*	Nerve_Tibial	1.47E-06	4.71E-03	0.197601
rs2535629	*TMEM110*	Whole_Blood	7.45E-06	1.96E-02	0.121372
rs2535629	*SFMBT1*	Esophagus_Gastroesophageal_Junction	1.57E-05	0.03718853876	0.245853
rs2535629	*SFMBT1*	Whole_Blood	8.72E-06	0.02250290887	0.147396
rs2535629	*GNL3*	Artery_Coronary	3.43E-06	9.97E-03	-0.174479
rs2535629	*GNL3*	Artery_Tibial	2.40E-07	0.0009223035167	-0.161703
rs2535629	*GNL3*	Brain_Cerebellum	4.82E-08	0.0002146692818	-0.447907
rs2535629	*GNL3*	Cells_Transformed_fibroblasts	1.64E-08	7.99E-05	-0.126615
rs2535629	*GNL3*	Esophagus_Muscularis	1.33E-06	0.004303076354	-0.171801
rs2535629	*GNL3*	Heart_Left_Ventricle	2.34E-07	0.0009018361762	-0.137947
rs2535629	*GNL3*	Nerve_Tibial	2.15E-07	0.000838242755	-0.126861
rs2535629	*GNL3*	Pituitary	3.46E-07	0.001282953661	-0.223075

rs2535629	*GNL3*	Testis	1.51E-07	6.10E-04	-0.204794
rs2535629	*PBRM1*	Artery_Tibial	3.67E-13	3.45E-09	0.158823
rs2535629	*PBRM1*	Cells_Transformed_fibroblasts	7.30E-06	1.93E-02	-0.113194
rs2535629	*PBRM1*	Thyroid	4.25E-29	1.92E-24	0.371498
rs2535629	*NT5DC2*	Cells_EBV-transformed_lymphocytes	2.45E-06	0.0073734478	-0.40891
rs2535629	*NT5DC2*	Cells_Transformed_fibroblasts	2.45E-07	9.41E-04	-0.104395
rs2535629	*NT5DC2*	Colon_Transverse	4.54E-08	2.03E-04	-0.249637
rs2535629	*NT5DC2*	Skin_Not_Sun_Exposed_Suprapubic	4.91E-09	2.62E-05	-0.188731
rs2535629	*NT5DC2*	Skin_Sun_Exposed_Lower_leg	2.51E-10	1.63E-06	-0.192675
rs2535629	*NT5DC2*	Thyroid	6.80E-11	4.78E-07	-0.226272
rs2535629	*GLYCTK*	Lung	1.93E-07	0.0007616221024	-0.175762
rs2535629	*GLYCTK*	Nerve_Tibial	2.18E-06	0.006650054194	-0.20308
rs2535629	*WDR82*	Cells_Transformed_fibroblasts	7.89E-06	0.02061497679	0.134094
rs2535629	*PPM1M*	Brain_Cerebellar_Hemisphere	6.56E-06	0.01758077668	-0.357333
rs2535629	*PPM1M*	Brain_Cerebellum	6.98E-07	0.002418972064	-0.396815
rs2535629	*RBMS3*	Muscle_Skeletal	2.13E-05	0.04808685455	0.1576839133
rs2535629	*RAF1*	Cells_EBV-transformed_lymphocytes	6.26E-06	0.01689737162	-0.3826631664
rs2535629	*ABCC1*	Brain_Cerebellar_Hemisphere	1.98E-05	0.04527864477	0.359997259
**Schizophrenia**					
rs2535629	*ITIH4*	Adipose_Subcutaneous	2.73E-10	2.75E-06	0.222223
rs2535629	*ITIH4*	Adipose_Visceral_Omentum	1.56E-06	0.006040987722	0.219241
rs2535629	*ITIH4*	Artery_Aorta	1.81E-19	8.13E-15	0.650073
rs2535629	*ITIH4*	Artery_Coronary	4.74E-09	3.65E-05	0.457006
rs2535629	*ITIH4*	Artery_Tibial	8.54E-20	4.04E-15	0.508774
rs2535629	*ITIH4*	Brain_Caudate_basal_ganglia	6.55E-06	0.02034162478	0.352338
rs2535629	*ITIH4*	Brain_Cerebellum	5.40E-07	0.002428264088	0.489369
rs2535629	*ITIH4*	Brain_Frontal_Cortex_BA9	1.08E-05	0.03079101209	0.416162
rs2535629	*ITIH4*	Brain_Hypothalamus	8.31E-07	0.003516328747	0.568006
rs2535629	*ITIH4*	Brain_Putamen_basal_ganglia	2.07E-06	0.007680122369	0.442893
rs2535629	*ITIH4*	Breast_Mammary_Tissue	1.63E-07	0.0008533100557	0.286252
rs2535629	*ITIH4*	Cells_EBV-transformed_lymphocytes	5.26E-07	0.002372399275	0.59652
rs2535629	*ITIH4*	Cells_Transformed_fibroblasts	1.14E-13	2.16E-09	0.444591
rs2535629	*ITIH4*	Colon_Transverse	7.14E-10	6.57E-06	0.249851
rs2535629	*ITIH4*	Esophagus_Mucosa	2.25E-12	3.34E-08	0.345726
rs2535629	*ITIH4*	Esophagus_Muscularis	4.90E-07	0.002234770458	0.188342
rs2535629	*ITIH4*	Heart_Atrial_Appendage	3.52E-11	4.18E-07	0.429801
rs2535629	*ITIH4*	Lung	4.96E-17	1.55E-12	0.401567
rs2535629	*ITIH4*	Muscle_Skeletal	9.48E-11	1.04E-06	0.182986
rs2535629	*ITIH4*	Nerve_Tibial	1.87E-12	2.82E-08	0.294419
rs2535629	*ITIH4*	Pituitary	2.84E-07	0.001387794363	0.518171
rs2535629	*ITIH4*	Skin_Not_Sun_Exposed_Suprapubic	1.33E-11	1.72E-07	0.310305
rs2535629	*ITIH4*	Skin_Sun_Exposed_Lower_leg	6.05E-14	1.20E-09	0.273844
rs2535629	*ITIH4*	Small_Intestine_Terminal_Ileum	4.99E-06	0.01618494633	0.294474
rs2535629	*ITIH4*	Thyroid	1.30E-19	5.95E-15	0.387306

rs2535629	*ITIH4*	Whole_Blood	4.27E-18	1.56E-13	0.340851
rs2535629	*NEK4*	Adipose_Visceral_Omentum	8.62E-06	0.02546263372	-0.187477
rs2535629	*NEK4*	Artery_Tibial	2.47E-06	0.008948686829	-0.185873
rs2535629	*NEK4*	Breast_Mammary_Tissue	8.21E-07	0.003480333187	-0.261104
rs2535629	*NEK4*	Cells_Transformed_fibroblasts	8.24E-07	0.003489807427	-0.201519
rs2535629	*NEK4*	Esophagus_Mucosa	1.95E-06	0.007305478171	-0.220788
rs2535629	*NEK4*	Heart_Atrial_Appendage	5.63E-07	0.00251550376	-0.245985
rs2535629	*NEK4*	Minor_Salivary_Gland	3.77E-06	0.01280012235	-0.476938
rs2535629	*NEK4*	Nerve_Tibial	1.22E-08	8.54E-05	-0.23281
rs2535629	*NEK4*	Skin_Sun_Exposed_Lower_leg	1.65E-08	0.0001116368377	-0.185539
rs2535629	*NEK4*	Thyroid	2.06E-07	0.00104616302	-0.196945
rs2535629	*MUSTN1*	Artery_Aorta	5.16E-13	8.65E-09	0.487663
rs2535629	*MUSTN1*	Whole_Blood	1.03E-06	0.004232274969	0.189607
rs2535629	*TMEM110*	Artery_Aorta	9.69E-09	6.95E-05	0.317929
rs2535629	*TMEM110*	Lung	1.45E-05	0.03892069018	0.184534
rs2535629	*TMEM110*	Muscle_Skeletal	1.11E-09	9.78E-06	0.20707
rs2535629	*TMEM110*	Nerve_Tibial	1.47E-06	0.005727442337	0.197601
rs2535629	*TMEM110*	Whole_Blood	7.45E-06	0.02262403478	0.121372
rs2535629	*SFMBT1*	Esophagus_Gastroesophageal_Junction	1.57E-05	0.04141321635	0.245853
rs2535629	*SFMBT1*	Whole_Blood	8.72E-06	0.02571155601	0.147396
rs2535629	*GNL3*	Artery_Coronary	3.43E-06	0.01179446593	-0.174479
rs2535629	*GNL3*	Artery_Tibial	2.40E-07	0.001194061352	-0.161703
rs2535629	*GNL3*	Brain_Cerebellum	4.82E-08	0.000290260164	-0.447907
rs2535629	*GNL3*	Cells_Transformed_fibroblasts	1.64E-08	0.0001115262283	-0.126615
rs2535629	*GNL3*	Esophagus_Muscularis	1.33E-06	0.005254836539	-0.171801
rs2535629	*GNL3*	Heart_Left_Ventricle	2.34E-07	0.001167548659	-0.137947
rs2535629	*GNL3*	Nerve_Tibial	2.15E-07	0.001086122691	-0.126861
rs2535629	*GNL3*	Pituitary	3.46E-07	0.001651054888	-0.223075
rs2535629	*GNL3*	Testis	1.51E-07	0.0007993773811	-0.204794
rs2535629	*PBRM1*	Artery_Tibial	3.67E-13	6.32E-09	0.158823
rs2535629	*PBRM1*	Cells_Transformed_fibroblasts	7.30E-06	0.02223904666	-0.113194
rs2535629	*PBRM1*	Thyroid	4.25E-29	5.38E-24	0.371498
rs2535629	*NT5DC2*	Cells_EBV-transformed_lymphocytes	2.45E-06	0.008866586318	-0.40891
rs2535629	*NT5DC2*	Cells_Transformed_fibroblasts	2.45E-07	0.001218631405	-0.104395
rs2535629	*NT5DC2*	Colon_Transverse	4.54E-08	0.0002756121279	-0.249637
rs2535629	*NT5DC2*	Skin_Not_Sun_Exposed_Suprapubic	4.91E-09	3.76E-05	-0.188731
rs2535629	*NT5DC2*	Skin_Sun_Exposed_Lower_leg	2.51E-10	2.54E-06	-0.192675
rs2535629	*NT5DC2*	Thyroid	6.80E-11	7.66E-07	-0.226272
rs2535629	*GLYCTK*	Lung	1.93E-07	0.0009890526783	-0.175762
rs2535629	*GLYCTK*	Nerve_Tibial	2.18E-06	0.008023857243	-0.20308
rs2535629	*WDR82*	Cells_Transformed_fibroblasts	7.89E-06	0.02369456807	0.134094
rs2535629	*PPM1M*	Brain_Cerebellar_Hemisphere	6.56E-06	0.02038224064	-0.357333
rs2535629	*PPM1M*	Brain_Cerebellum	6.98E-07	0.003027637044	-0.396815
rs2535629	*RAF1*	Cells_EBV-transformed_lymphocytes	6.26E-06	0.01957318639	-0.3826631664

rs2535629	*ABCC1*	Brain_Cerebellar_Hemisphere	1.98E-05	0.04997276634	0.359997259

Supplementary Table 5. Unique and shared pathways between psychiatric disorders and cognitive functioning. "yes" indicates the association of the pathway with phenotype.

№	iPathway	ADHD	Anx	BD	UD	SCZ	Cognition
1	Acute myeloid leukemia	yes	yes	yes	yes	yes	yes
2	Adrenergic signaling in cardiomyocytes	yes	yes	yes	yes	yes	yes
3	Alcoholism	yes	yes	yes	yes	yes	yes
4	Antigen processing and presentation	yes	yes	yes	yes	yes	yes
5	Apelin signaling pathway	yes	yes	yes	yes	yes	yes
6	Autophagy - animal	yes	yes	yes	yes	yes	yes
7	Axon guidance	yes	yes	yes	yes	yes	yes
8	Breast cancer	yes	yes	yes	yes	yes	yes
9	Cellular senescence	yes	yes	yes	yes	yes	yes
10	Chemokine signaling pathway	yes	yes	yes	yes	yes	yes
11	Chronic myeloid leukemia	yes	yes	yes	yes	yes	yes
12	Colorectal cancer	yes	yes	yes	yes	yes	yes
13	EGFR tyrosine kinase inhibitor resistance	yes	yes	yes	yes	yes	yes
14	Endocrine resistance	yes	yes	yes	yes	yes	yes
15	Endometrial cancer	yes	yes	yes	yes	yes	yes
16	Fc gamma R-mediated phagocytosis	yes	yes	yes	yes	yes	yes
17	Fluid shear stress and atherosclerosis	yes	yes	yes	yes	yes	yes
18	Focal adhesion	yes	yes	yes	yes	yes	yes
19	FoxO signaling pathway	yes	yes	yes	yes	yes	yes
20	Gastric cancer	yes	yes	yes	yes	yes	yes
21	Glioma	yes	yes	yes	yes	yes	yes
22	GnRH signaling pathway	yes	yes	yes	yes	yes	yes
23	Hepatitis B	yes	yes	yes	yes	yes	yes
24	Hepatitis C	yes	yes	yes	yes	yes	yes
25	Hepatocellular carcinoma	yes	yes	yes	yes	yes	yes
26	Hippo signaling pathway	yes	yes	yes	yes	yes	yes
27	Human cytomegalovirus infection	yes	yes	yes	yes	yes	yes
28	Human immunodeficiency virus 1 infection	yes	yes	yes	yes	yes	yes

29	Human papillomavirus infection	yes	yes	yes	yes	yes	yes
30	Influenza A	yes	yes	yes	yes	yes	yes
31	Insulin resistance	yes	yes	yes	yes	yes	yes
32	Insulin signaling pathway	yes	yes	yes	yes	yes	yes
33	Kaposi sarcoma-associated herpesvirus infection	yes	yes	yes	yes	yes	yes
34	Long-term depression	yes	yes	yes	yes	yes	yes
35	Long-term potentiation	yes	yes	yes	yes	yes	yes
36	MAPK signaling pathway	yes	yes	yes	yes	yes	yes
37	Melanogenesis	yes	yes	yes	yes	yes	yes
38	Melanoma	yes	yes	yes	yes	yes	yes
39	Natural killer cell mediated cytotoxicity	yes	yes	yes	yes	yes	yes
40	Neurotrophin signaling pathway	yes	yes	yes	yes	yes	yes
41	Non-small cell lung cancer	yes	yes	yes	yes	yes	yes
42	PD-L1 expression and PD-1 checkpoint pathway in cancer	yes	yes	yes	yes	yes	yes
43	PI3K-Akt signaling pathway	yes	yes	yes	yes	yes	yes
44	Parathyroid hormone synthesis, secretion and action	yes	yes	yes	yes	yes	yes
45	Pathways in cancer	yes	yes	yes	yes	yes	yes
46	Phospholipase D signaling pathway	yes	yes	yes	yes	yes	yes
47	Prolactin signaling pathway	yes	yes	yes	yes	yes	yes
48	Prostate cancer	yes	yes	yes	yes	yes	yes
49	Proteoglycans in cancer	yes	yes	yes	yes	yes	yes
50	Rap1 signaling pathway	yes	yes	yes	yes	yes	yes
51	Ras signaling pathway	yes	yes	yes	yes	yes	yes
52	Relaxin signaling pathway	yes	yes	yes	yes	yes	yes
53	TGF-beta signaling pathway	yes	yes	yes	yes	yes	yes
54	Thyroid hormone signaling pathway	yes	yes	yes	yes	yes	yes
55	Tight junction	yes	yes	yes	yes	yes	yes
56	Tuberculosis	yes	yes	yes	yes	yes	yes
57	Vascular smooth muscle contraction	yes	yes	yes	yes	yes	yes
58	Wnt signaling pathway	yes	yes	yes	yes	yes	yes
59	cAMP signaling pathway	yes	yes	yes	yes	yes	yes
60	cGMP-PKG signaling pathway	yes	yes	yes	yes	yes	yes
61	mTOR signaling pathway	yes	yes	yes	yes	yes	yes

62	Estrogen signaling pathway	yes	yes	yes	yes	yes	
63	Gap junction	yes	yes	yes	yes	yes	
64	Notch signaling pathway	yes	yes	yes	yes	yes	
65	Olfactory transduction	yes	yes	yes	yes	yes	
66	Vibrio cholerae infection	yes	yes	yes	yes	yes	
67	Aldosterone synthesis and secretion	yes	yes		yes	yes	yes
68	Bile secretion	yes	yes				
69	Apoptosis	yes		yes	yes	yes	yes
70	B cell receptor signaling pathway	yes		yes	yes	yes	yes
71	C-type lectin receptor signaling pathway	yes		yes	yes	yes	yes
72	ErbB signaling pathway	yes		yes	yes	yes	yes
73	Fc epsilon RI signaling pathway	yes		yes	yes	yes	yes
74	Inflammatory bowel disease (IBD)	yes		yes	yes	yes	yes
75	Oxytocin signaling pathway	yes		yes	yes	yes	yes
76	Pancreatic cancer	yes		yes	yes	yes	yes
77	Pertussis	yes		yes	yes	yes	yes
78	Serotonergic synapse	yes		yes	yes	yes	yes
79	Sphingolipid signaling pathway	yes		yes	yes	yes	yes
80	Staphylococcus aureus infection	yes		yes	yes	yes	yes
81	Systemic lupus erythematosus	yes		yes	yes	yes	yes
82	T cell receptor signaling pathway	yes		yes	yes	yes	yes
83	Th1 and Th2 cell differentiation	yes		yes	yes	yes	yes
84	Th17 cell differentiation	yes		yes	yes	yes	yes
85	VEGF signaling pathway	yes		yes	yes	yes	yes
86	Bladder cancer	yes		yes	yes	yes	
87	Central carbon metabolism in cancer	yes		yes	yes	yes	
88	Choline metabolism in cancer	yes		yes	yes	yes	
89	Renal cell carcinoma	yes		yes	yes	yes	
90	African trypanosomiasis	yes			yes	yes	
91	Amoebiasis	yes			yes	yes	
92	Pancreatic secretion	yes					
93	AGE-RAGE signaling pathway in diabetic complications		yes	yes	yes	yes	yes
94	AMPK signaling pathway		yes	yes	yes	yes	yes

95	Adipocytokine signaling pathway		yes	yes	yes	yes	yes
96	Alzheimer disease		yes	yes	yes	yes	yes
97	Amphetamine addiction		yes	yes	yes	yes	yes
98	Calcium signaling pathway		yes	yes	yes	yes	yes
99	Cell cycle		yes	yes	yes	yes	yes
100	Cocaine addiction		yes	yes	yes	yes	yes
101	Cushing syndrome		yes	yes	yes	yes	yes
102	Dopaminergic synapse		yes	yes	yes	yes	yes
103	Endocrine and other factor-regulated calcium reabsorption		yes	yes	yes	yes	yes
104	Glucagon signaling pathway		yes	yes	yes	yes	yes
105	HIF-1 signaling pathway		yes	yes	yes	yes	yes
106	Herpes simplex virus 1 infection		yes	yes	yes	yes	yes
107	Human T-cell leukemia virus 1 infection		yes	yes	yes	yes	yes
108	Leishmaniasis		yes	yes	yes	yes	yes
109	Longevity regulating pathway		yes	yes	yes	yes	yes
110	Longevity regulating pathway - multiple species		yes	yes	yes	yes	yes
111	Non-alcoholic fatty liver disease (NAFLD)		yes	yes	yes	yes	yes
112	Oocyte meiosis		yes	yes	yes	yes	yes
113	Platelet activation		yes	yes	yes	yes	yes
114	Regulation of actin cytoskeleton		yes	yes	yes	yes	yes
115	Thermogenesis		yes	yes	yes	yes	yes
116	Toxoplasmosis		yes	yes	yes	yes	yes
117	Progesterone-mediated oocyte maturation		yes	yes		yes	
118	Basal cell carcinoma		yes		yes	yes	yes
119	Cholinergic synapse		yes		yes	yes	yes
120	Circadian entrainment		yes		yes	yes	yes
121	Cortisol synthesis and secretion		yes		yes	yes	yes
122	Hedgehog signaling pathway		yes		yes	yes	yes
123	Hippo signaling pathway - multiple species		yes		yes	yes	yes
124	Insulin secretion		yes		yes	yes	yes
125	JAK-STAT signaling pathway		yes		yes	yes	yes
126	Morphine addiction		yes		yes	yes	yes
127	Necroptosis		yes		yes	yes	yes

128	Regulation of lipolysis in adipocytes		yes		yes	yes	yes
129	Salivary secretion		yes		yes	yes	yes
130	Thyroid hormone synthesis		yes		yes	yes	yes
131	Renin secretion		yes		yes	yes	
132	Retrograde endocannabinoid signaling		yes		yes	yes	
133	Gastric acid secretion		yes		yes		yes
134	Phototransduction		yes		yes		yes
135	RNA transport		yes		yes		
136	Viral carcinogenesis		yes		yes		
137	Thyroid cancer		yes				yes
138	Viral myocarditis		yes				yes
139	Dilated cardiomyopathy (DCM)		yes				
140	Ovarian steroidogenesis		yes				
141	Vasopressin-regulated water reabsorption		yes				
142	Bacterial invasion of epithelial cells			yes	yes	yes	yes
143	Chagas disease (American trypanosomiasis)			yes	yes	yes	yes
144	Circadian rhythm			yes	yes	yes	yes
145	Cytokine-cytokine receptor interaction			yes	yes	yes	yes
146	Epstein-Barr virus infection			yes	yes	yes	yes
147	Glutamatergic synapse			yes	yes	yes	yes
148	Measles			yes	yes	yes	yes
149	NF-kappa B signaling pathway			yes	yes	yes	yes
150	NOD-like receptor signaling pathway			yes	yes	yes	yes
151	Osteoclast differentiation			yes	yes	yes	yes
152	Shigellosis			yes	yes	yes	yes
153	Small cell lung cancer			yes	yes	yes	yes
154	Epithelial cell signaling in Helicobacter pylori infection			yes	yes	yes	
155	Toll-like receptor signaling pathway			yes	yes	yes	
156	TNF signaling pathway			yes	yes		
157	PPAR signaling pathway			yes		yes	yes
158	Platinum drug resistance			yes		yes	
159	Aldosterone-regulated sodium reabsorption			yes			yes
160	Fanconi anemia pathway			yes			yes
161	RIG-I-like receptor signaling pathway			yes			yes

162	Antifolate resistance			yes			
163	Cytosolic DNA-sensing pathway			yes			
164	IL-17 signaling pathway			yes			
165	Complement and coagulation cascades				yes	yes	yes
166	ECM-receptor interaction				yes	yes	yes
167	Leukocyte transendothelial migration				yes	yes	yes
168	Mitophagy - animal				yes	yes	yes
169	Neuroactive ligand-receptor interaction				yes	yes	yes
170	Pathogenic Escherichia coli infection				yes	yes	yes
171	Protein processing in endoplasmic reticulum				yes	yes	yes
172	Salmonella infection				yes	yes	yes
173	Type II diabetes mellitus				yes	yes	yes
174	Amyotrophic lateral sclerosis (ALS)				yes	yes	
175	GABAergic synapse				yes	yes	
176	Legionellosis				yes	yes	
177	Autophagy - other				yes		yes
178	Taste transduction				yes		yes
179	Malaria				yes		
180	Rheumatoid arthritis				yes		
181	Prion diseases					yes	
182	p53 signaling pathway					yes	
183	Carbohydrate digestion and absorption						yes

Supplementary Table 6. Unique and shared brain-specific pathways between psychiatric disorders and cognitive functioning. “yes” indicates the association of the pathway with phenotype.

№	iPathway	ADHD	Anx	BD	UD	SCZ	Cognition
1	Natural killer cell mediated cytotoxicity	yes		yes	yes	yes	yes
2	Th1 and Th2 cell differentiation	yes		yes	yes	yes	yes
3	Antigen processing and presentation	yes		yes	yes	yes	
4	Pertussis	yes		yes	yes	yes	
5	Staphylococcus aureus infection	yes		yes	yes	yes	
6	Systemic lupus erythematosus	yes		yes	yes	yes	
7	Cellular senescence	yes			yes	yes	yes
8	Pathways in cancer		yes	yes		yes	yes

9	Rap1 signaling pathway		yes		yes	yes	yes
10	Vascular smooth muscle contraction		yes		yes	yes	
11	MAPK signaling pathway		yes		yes		yes
12	PI3K-Akt signaling pathway		yes		yes		yes
13	Parathyroid hormone synthesis, secretion and action		yes		yes		
14	Phospholipase D signaling pathway		yes		yes		
15	Hippo signaling pathway		yes			yes	yes
16	Colorectal cancer		yes			yes	
17	JAK-STAT signaling pathway		yes			yes	
18	Prostate cancer		yes			yes	
19	Calcium signaling pathway		yes				yes
20	Breast cancer		yes				
21	Chronic myeloid leukemia		yes				
22	Endocrine and other factor-regulated calcium reabsorption		yes				
23	Endometrial cancer		yes				
24	Gastric cancer		yes				
25	Hepatocellular carcinoma		yes				
26	Melanogenesis		yes				
27	Thyroid cancer		yes				
28	Wnt signaling pathway		yes				
29	Axon guidance			yes	yes	yes	yes
30	Focal adhesion			yes	yes	yes	yes
31	Proteoglycans in cancer			yes	yes	yes	yes
32	Regulation of actin cytoskeleton			yes	yes	yes	yes
33	Platelet activation			yes	yes	yes	
34	Small cell lung cancer			yes		yes	yes
35	Bacterial invasion of epithelial cells			yes		yes	
36	Human papillomavirus infection			yes			
37	Notch signaling pathway			yes			
38	Autophagy - animal				yes	yes	yes
39	Human cytomegalovirus infection				yes	yes	yes
40	Ras signaling pathway				yes	yes	yes
41	Tight junction				yes	yes	yes
42	C-type lectin receptor signaling pathway				yes	yes	
43	Chemokine signaling pathway				yes	yes	
44	Hepatitis B				yes	yes	
45	Human T-cell leukemia virus 1 infection				yes	yes	

46	Insulin resistance				yes	yes	
47	Insulin signaling pathway				yes	yes	
48	Leukocyte transendothelial migration				yes	yes	
49	Sphingolipid signaling pathway				yes	yes	
50	VEGF signaling pathway				yes	yes	
51	cAMP signaling pathway				yes	yes	
52	mTOR signaling pathway				yes	yes	
53	FoxO signaling pathway				yes		yes
54	PD-L1 expression and PD-1 checkpoint pathway in cancer				yes		yes
55	Th17 cell differentiation				yes		yes
56	cGMP-PKG signaling pathway				yes		yes
57	AMPK signaling pathway				yes		
58	Adipocytokine signaling pathway				yes		
59	Apelin signaling pathway				yes		
60	Circadian rhythm				yes		
61	Glucagon signaling pathway				yes		
62	Kaposi sarcoma-associated herpesvirus infection				yes		
63	Long-term depression				yes		
64	Longevity regulating pathway				yes		
65	Longevity regulating pathway - multiple species				yes		
66	Oxytocin signaling pathway				yes		
67	Salmonella infection				yes		
68	T cell receptor signaling pathway				yes		
69	Toxoplasmosis				yes		
70	Apoptosis					yes	yes
71	Cell cycle					yes	yes
72	Epstein-Barr virus infection					yes	yes
73	Fc epsilon RI signaling pathway					yes	yes
74	Fluid shear stress and atherosclerosis					yes	yes
75	Human immunodeficiency virus 1 infection					yes	yes
76	Non-alcoholic fatty liver disease (NAFLD)					yes	yes
77	Non-small cell lung cancer					yes	yes
78	Thyroid hormone signaling pathway					yes	yes
79	AGE-RAGE signaling pathway in diabetic complications					yes	
80	Central carbon metabolism in cancer					yes	
81	Chagas disease (American trypanosomiasis)					yes	
82	Choline metabolism in cancer					yes	

83	Cholinergic synapse					yes	
84	Complement and coagulation cascades					yes	
85	Cortisol synthesis and secretion					yes	
86	Cushing syndrome					yes	
87	Endocrine resistance					yes	
88	Estrogen signaling pathway					yes	
89	Fc gamma R-mediated phagocytosis					yes	
90	GnRH signaling pathway					yes	
91	HIF-1 signaling pathway					yes	
92	Melanoma					yes	
93	Mitophagy - animal					yes	
94	NOD-like receptor signaling pathway					yes	
95	Neuroactive ligand-receptor interaction					yes	
96	Neurotrophin signaling pathway					yes	
97	Osteoclast differentiation					yes	
98	Platinum drug resistance					yes	
99	Regulation of lipolysis in adipocytes					yes	
100	Relaxin signaling pathway					yes	
101	Toll-like receptor signaling pathway					yes	
102	Tuberculosis					yes	
103	Type II diabetes mellitus					yes	
104	ErbB signaling pathway						yes
105	Herpes simplex virus 1 infection						yes
106	Hippo signaling pathway - multiple species						yes
107	Inflammatory bowel disease (IBD)						yes
108	Leishmaniasis						yes
109	NF-kappa B signaling pathway						yes
110	Protein processing in endoplasmic reticulum						yes
